# Interfacial chemistry of sulfide and halide solid electrolytes in all solid-state sodium batteries: from single electrolytes to functionally partitioned architectures

**DOI:** 10.1039/d6sc05921j

**Published:** 2026-09-08

**Authors:** Lin Li, Wenqian Tian, Siwu Li, Miao Deng, Ziyu Lu, Chuang Yu

**Affiliations:** a School of Information Mechanics and Sensing Engineering, Xidian University Xi'an Shaanxi 710126 P. R. China lisiwu@xidian.edu.cn yuchuang@xidian.edu.cn; b School of Chemistry and Chemical Engineering, Huazhong University of Science and Technology Wuhan 430074 P. R. China cyu2020@hust.edu.cn

## Abstract

All solid-state sodium batteries (ASSSBs) are promising for safe, low cost, and large-scale energy storage, but further development is limited by the difficulty of identifying an inorganic solid electrolyte (SE) that remains stable against both high voltage cathodes and low potential sodium metal anodes. This review focuses on sulfide and halide solid electrolytes, two representative sodium based inorganic electrolyte families with complementary properties. Sulfides provide high room-temperature sodium-ion conductivity, good cold pressed contact, and efficient ion transport networks, mainly because sulfide anions are highly polarizable and form soft lattices. The same features, however, raise the valence band maximum and make sulfides prone to oxidative decomposition at high-voltage cathode interfaces. Halide electrolytes are generally more stable against oxidation and more compatible with oxide cathodes because of their deeper valence bands. However, the low lying conduction bands and reducible high valent metal cations in many halide electrolytes can lead to reduction reactions at the Na metal interface. Sulfide electrolytes are also thermodynamically unstable against Na metal. In some cases, however, their interfacial reactions can be kinetically suppressed by artificial interlayers that limit electron transfer and relieve interfacial stress. This review summarizes the structural chemistry, transport mechanisms, defect regulation, cathode oxidation behavior, anode reduction behavior, and interface engineering of sulfide and halide electrolytes. It then discusses cathode passivation, artificial anode interlayers, and sulfide–halide heterointerface compatibility. Finally, we propose functionally partitioned electrolyte architectures as an emerging design framework that assigns ion transport, oxidation resistance, reduction protection, and mechanical accommodation to distinct yet compatible electrolyte components. Although current studies in sodium systems provide preliminary evidence of feasibility, systematic investigation of the interfacial chemistry and Na ion transport across heterointerfaces between sulfide and halide electrolytes remains limited.

## Abstract 2

For sulfide and halide Na electrolytes, sulfides conduct but oxidize, halides resist oxidation but reduce. Here we review their structures, interfaces, and propose partitioned designs separating ion transport, redox protection, and mechanics.

## Graphical abstract

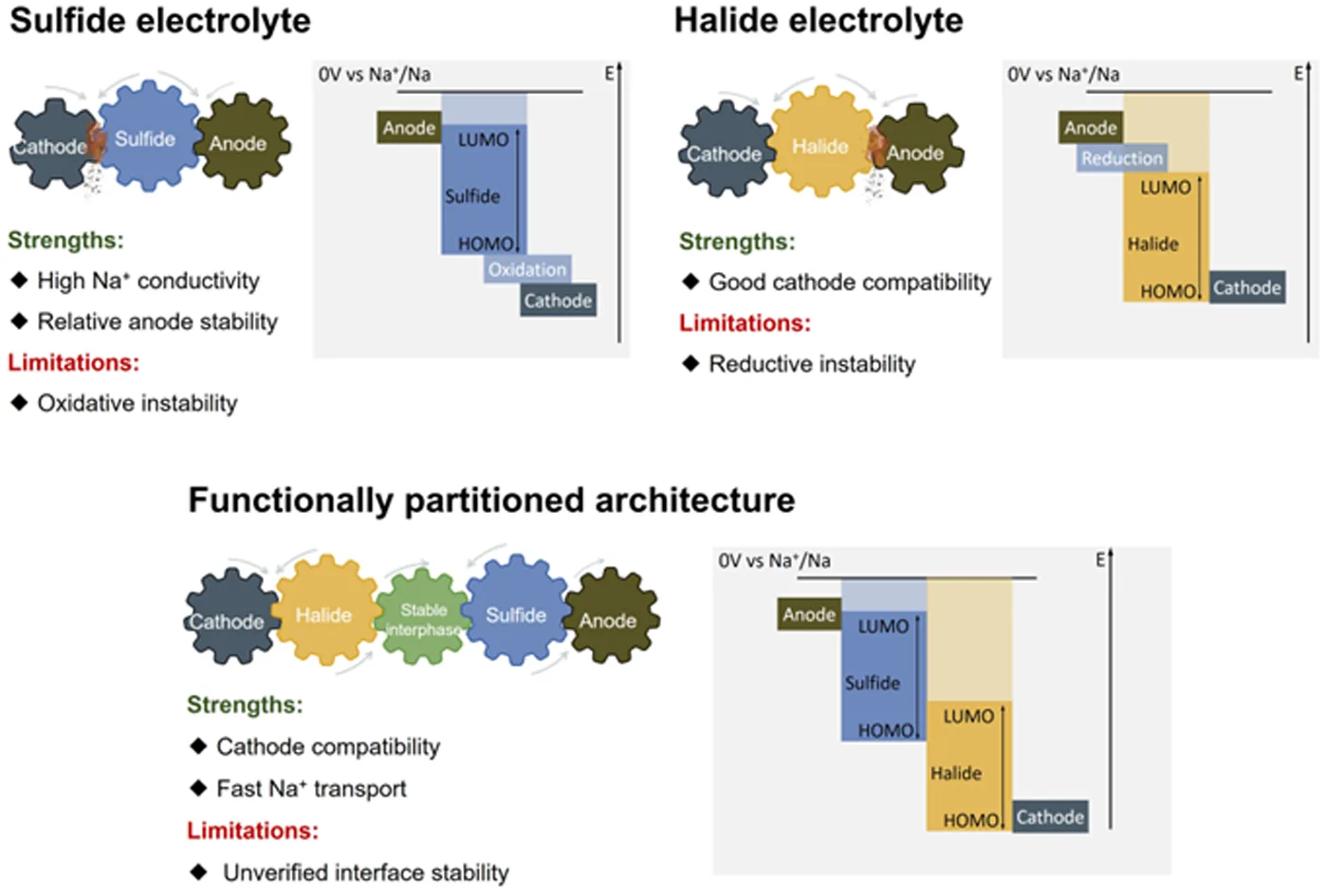

## 1. Introduction

Sodium-ion batteries are widely regarded as a promising technology for large scale electrochemical energy storage because of the high abundance of sodium in the Earth's crust (2.74%), its relatively uniform global distribution, and the low cost of sodium resources.^1–3^ Replacing organic liquid electrolytes with inorganic solid-state electrolytes to assemble all-solid-state sodium batteries (ASSSBs) can not only mitigate the flammability and leakage risks associated with liquid electrolytes but also help overcome the energy density limitations of conventional sodium ion batteries by enabling the possible use of Na metal anodes.^3–8^ However, among the reported inorganic solid electrolytes (SEs) for sodium batteries, it remains challenging to identify a single homogeneous electrolyte that can maintain satisfactory interfacial stability under both the strong reducing environment of a Na metal anode (0 V *vs.* Na^+^/Na) and the strongly oxidizing environment of a high voltage oxide cathode (>4 V *vs.* Na^+^/Na).^7,9–11^ This dilemma arises from the difficulty of simultaneously optimizing ionic conductivity, cathode side oxidation resistance, and anode side reduction resistance within a single-anion framework.^12–17^ This compatibility bottleneck directly limits the energy density and cycle life of ASSSBs.

In ASSSBs, reported inorganic SEs include oxides, sulfides, halides, borohydrides, and polymer SEs. Existing reviews have mainly discussed these systems by electrolyte family, transport mechanism, or individual interface problems, and have clarified the material specific merits of different SEs.^8,18^ However, a unified framework connecting electrolyte chemistry with the spatial allocation of functions in a complete cell remains insufficient. In particular, it remains unclear how to select and combine electrolytes that must simultaneously support fast Na^+^ transport, resist oxidation at high voltage cathodes, and resist reduction at Na metal anodes. Sulfides and halides are therefore considered together in this review, not simply because they are two important inorganic electrolyte families, but because they represent complementary solutions to the same compatibility bottleneck. Sulfides typically offer high room temperature Na^+^ conductivity and favorable cold pressing contact, whereas halides generally provide stronger cathode side oxidation resistance and better compatibility with high voltage oxide cathodes.^19^ Meanwhile, sulfide electrolytes are more prone to oxidation at the cathode side, whereas many halide electrolytes undergo more serious reduction reactions at the Na metal side. At low potentials, neither sulfide nor halide electrolytes are thermodynamically stable against Na metal. Instead, their different decomposition reactions require different interface design strategies. Comparing them within one framework makes it possible to link anion electronic structure with ion transport, electrochemical stability, mechanical processability, and interfacial failure. On this basis, this review examines functionally partitioned electrolyte architectures in which cathode protection, bulk ion transport, anode protection, and mechanical buffering are assigned to different but compatible components. This architecture is treated as a design framework for decoupling competing requirements, rather than as a universally validated solution.

Within this tradeoff spectrum, sulfides use S^2−^ as the core anion, which is characterized by a large ionic radius and low electronegativity. Compared with O^2−^, the larger ionic radius of S^2−^ provides greater lattice free volume and wider Na^+^ migration channels, while its lower electronegativity gives the S^2−^ electron cloud higher polarizability, enabling dynamic screening of coulombic repulsion as Na^+^ passes through geometric bottlenecks.^16,20^ These two effects jointly lower the Na^+^ migration barrier, leading to activation energies that typically fall within 0.15 to 0.40 eV for sulfide electrolytes.^21–23^ The room-temperature conductivity of Na_3_SbS_4_ (NSS) can reach 3 mS cm^−1^ and can be further increased to over 10 mS cm^−1^ by W doping.^24–29^ The Na_3_PS_4_ system can reach a room-temperature conductivity of 26.4 mS cm^−1^ after W doping and precursor purification.^30^ High-entropy sulfide Na_3−*x*_Sb_1−4*x*_(SnWCaTi)_*x*_S_4_ also achieves a room-temperature conductivity of 6.3 mS cm^−1^.^31^ In terms of processability, sulfides can form large contact areas with cathode particles through simple cold pressing, and some materials, such as Na_3_SbS_4_, also show good solution processability.^32,33^ However, despite their high ionic conductivity, the high energy S 3p derived valence band limits their intrinsic oxidative stability, with oxidation decomposition often occurring below approximately 2.5 V.^34–36^

Unlike sulfides, which rely on high anion polarizability and low migration barriers, chloride-based halides provide a different design logic. Cl^−^ has higher electronegativity and lower polarizability, which lowers the valence band maximum of halides and thereby broadens their electrochemical stability window. Their oxidation onset potentials can typically reach 3.8 V or higher, making them thermodynamically more compatible with layered oxide and polyanionic cathodes.^13,37,38^ However, early crystalline halides generally exhibited low room-temperature (RT) ionic conductivity, mainly because of limited Na^+^ site concentration, insufficient connectivity of migration pathways, and constraints imposed by the local coordination environment in relatively rigid lattices. In some systems, the conductivity remained in the range of 10^−6^ to 10^−5^ S cm^−1^ for a long time.^38–40^ To overcome this structural bottleneck, recent studies have reconstructed Na^+^ transport pathways through amorphization and oxychloride structural modulation. For example, the RT conductivity of the Na_2_O and TaCl_5_ glass system reached 4.62 mS cm^−1^,^41^ quenched NaTaOCl_4_ achieved 7.2 mS cm^−1^,^42^ and a carbonate and oxychloride composite reached 5.01 mS cm^−1^ after only 10 min of ball milling.^43^ These results indicate that halides are not intrinsically restricted to low conductivity. Instead, their Na^+^ transport potential can be released through disordering, defect regulation, and local coordination reconstruction. Furthermore, halides typically exhibit a low Young's modulus and high deformability, with crystalline halides showing Young's moduli of approximately 10 to 22 GPa. This mechanical feature facilitates conformal contact with cathode particle surfaces under cold pressing, enabling a continuous and dense ionic percolation network and improving interparticle contact within composite cathodes while maintaining high voltage stability.^44–46^

More importantly, sulfides and halides show a clear complementarity in cathode and anode interfacial stability. In sulfides, the high lying S 3p valence band and strong anion polarizability lower the Na^+^ migration barrier, but also render them vulnerable to oxidative decomposition at high potential cathodes. In oxide cathode composite layers, S^2−^ can be oxidized to S^0^ or polysulfides, increasing interfacial impedance and limiting capacity utilization.^47,48^ By contrast, sulfide electrolytes also undergo thermodynamically favorable reduction upon direct contact with Na metal. The resulting interphase can have different effects on further reactions. In favorable cases, a thin and electronically insulating interphase can limit electron transfer and slow further reduction. In unfavorable cases, electronically conductive or mixed ionic and electronic conducting products, such as Na_3_P, Na_3_Sb, or metallic species, can sustain interfacial reduction and lead to continuous electrolyte decomposition. Therefore, sulfide electrolytes should not be regarded as intrinsically stable against Na metal. Compared with many halide electrolytes, some sulfide electrolytes may be more readily stabilized by artificial interlayers, alloy modification, or controlled passivation.^49,50^

Halides exhibit the opposite stability profile. The low-lying Cl derived valence band provides higher oxidative stability at the cathode side, making halides more suitable for coupling with layered oxide and polyanionic cathodes. However, halides generally suffer from poor reduction stability against Na metal anodes. Many halides contain high valent metal cations, such as Zr^4+^ and Ta^5+^, whose low energy empty d orbitals can readily accept electrons injected from Na metal and be reduced to lower valent or even metallic species. If the resulting lower valent metal or metallic interfacial products (*e.g.*, NaCl) exhibit mixed ionic and electronic conduction, electrons can continue to penetrate through the reaction layer and drive progressive halide decomposition into the electrolyte interior.^51,52^ Direct comparative experiments by Goodwin *et al.* further confirmed this cathode side advantage of halides: when Na_0.67_Fe_0.33_Mn_0.67_O_2_ was used as the cathode, the Na_3_SbS_4_ composite cathode delivered an initial capacity of only 7 to 15 mAh g^−1^ with an overpotential as high as 1.5 V, whereas the Na_2.4_Er_0.4_Zr_0.6_Cl_6_ (NEZC) halide composite cathode exhibited a reversible capacity close to the theoretical value.^53^

These contrasting interfacial responses provide the conceptual basis for spatially partitioning electrolyte functions (Fig. 1). As illustrated schematically, sulfides offer fast Na^+^ transport but may undergo oxidative degradation at cathode interfaces (Fig. 1a), whereas halides provide better cathode compatibility but remain vulnerable to reduction near Na metal (Fig. 1b). A functionally partitioned architecture could therefore position a halide near the cathode and a sulfide toward the anode side, where the sulfide reduction interface would require adequate control (Fig. 1c). A regulated interphase between the two SEs could mitigate chemical incompatibility and support continuous Na^+^ transport. This configuration should be viewed as a proposed design framework rather than a universally validated architecture because direct evidence for the chemistry, stability, and transport properties of heterointerfaces between sodium sulfide and sodium halide SEs remains limited. Against this background, this review examines functionally partitioned electrolyte architectures. Sections 2 and 3 summarize the structural characteristics, ion transport mechanisms, mechanical processability, and cathode and anode interfacial behaviors of sulfide and halide SEs, respectively. Section 4 then discusses interface engineering strategies, including cathode interfacial passivation, composite cathode microstructure optimization, artificial anode interphase construction, and regulation of heterointerfaces between sulfide and halide SEs. Finally, the review evaluates functionally partitioned electrolyte architectures as an emerging design framework and identifies the evidence needed to establish their broader validity.

**Fig. 1:**
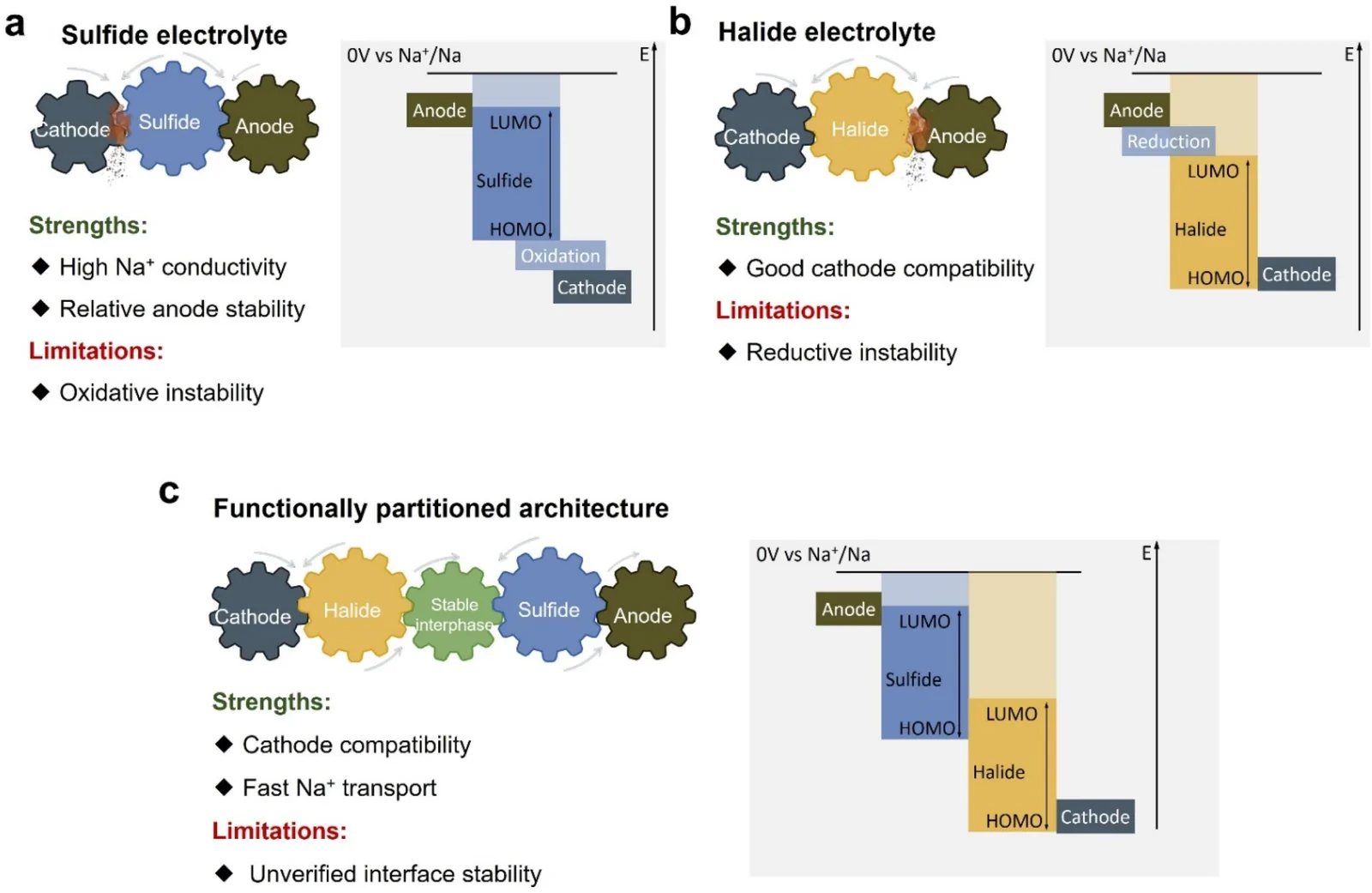
Schematic illustration of interfacial compatibility in ASSSBs. (a) Sulfide SEs are prone to oxidation at oxide cathodes and are thermodynamically reactive with anode. Continued reduction may be kinetically limited only when the resulting or deliberately introduced interphase provides sufficient electronic insulation and mechanical integrity. (b) Halide SEs generally provide better cathode side oxidation resistance but are also vulnerable to reduction near anode. (c) A functionally partitioned architecture places a halide near the cathode and uses an intentionally regulated artificial anode interlayer to protect the sulfide transport layer from direct anode contact.

## 2. Sulfide solid electrolytes

Among known sodium ion conductors, sulfides can achieve exceptionally high RT ionic conductivity because of the high polarizability and soft Lewis basicity of S^2−^, with conductivities approaching or even exceeding those of liquid electrolytes. However, the same anion electronic structure also raises the valence band maximum, making sulfides more susceptible to thermodynamic oxidative decomposition at high-voltage cathodes. The central challenge for sulfides is therefore not merely one of interface engineering, but arises from an intrinsic chemical tradeoff between their favorable ion transport properties and electrochemical stability, a balance that is difficult to optimize simultaneously. Starting from this contradiction, this section analyzes the conduction mechanisms of sulfide polymorphs, the limits of defect based chemical regulation, and the distinct interfacial failure modes at the anode and cathode sides, with the aim of defining the performance boundaries of pure sulfide systems.

### 2.1 Crystal chemistry and transport mechanisms underlying fast Na ion conduction in sulfides

The development of SEs with high room-temperature ionic conductivity, typically above 10^−3^ S cm^−1^, is a key prerequisite for realizing high performance RT ASSSBs. Among currently known inorganic SEs, sulfide materials generally exhibit Na^+^ transport kinetics superior to those of halides, oxides, polymers, and most borohydrides.^23,54^ According to Arrhenius-type ion-transport behavior, macroscopic ionic conductivity in a solid crystal lattice is jointly governed by carrier concentration, attempt frequency, and migration activation energy. Sulfide SEs can generally reduce the Na^+^ migration activation energy to 0.2 to 0.4 eV, and in some superionic conductors, it can even fall below 0.15 eV. The origin of this low migration barrier lies in the intrinsic physicochemical properties of the S^2−^ anion.^31,55–58^

Early research on sodium sulfide SEs can be traced back to the chemical strategy of replacing O^2−^ with S^2−^ in Na_3_PO_4_ derived frameworks. Compared with O^2−^, which has high electronegativity (Pauling electronegativity 3.44) and a small ionic radius (1.40 Å), S^2−^ has a larger ionic radius (approximately 1.84 Å) and lower electronegativity (2.58).^20,23,59^ This underlying physical difference gives rise to two crystallographic effects. First, the larger anion expands the lattice framework, providing greater free volume, spacious interstitial sites, and long range transport channels for Na^+^.^60^ Compared with Na_3_PO_4_, Na_3_PS_4_ shows a significantly expanded unit cell volume, thereby widening the ion migration bottleneck (Fig. 2a). Second, the highly deformable electron cloud of S^2−^ gives it high polarizability and soft base character, which not only weakens the electrostatic binding between Na^+^ and the S based framework but also dynamically screens surrounding coulombic repulsion as Na^+^ traverses geometric bottlenecks, thereby flattening the global potential energy surface for Na^+^ migration. Together, these two intrinsic factors provide the crystal chemical basis for the higher ionic conductivity of sulfides compared with oxides.^60,61^

**Fig. 2:**
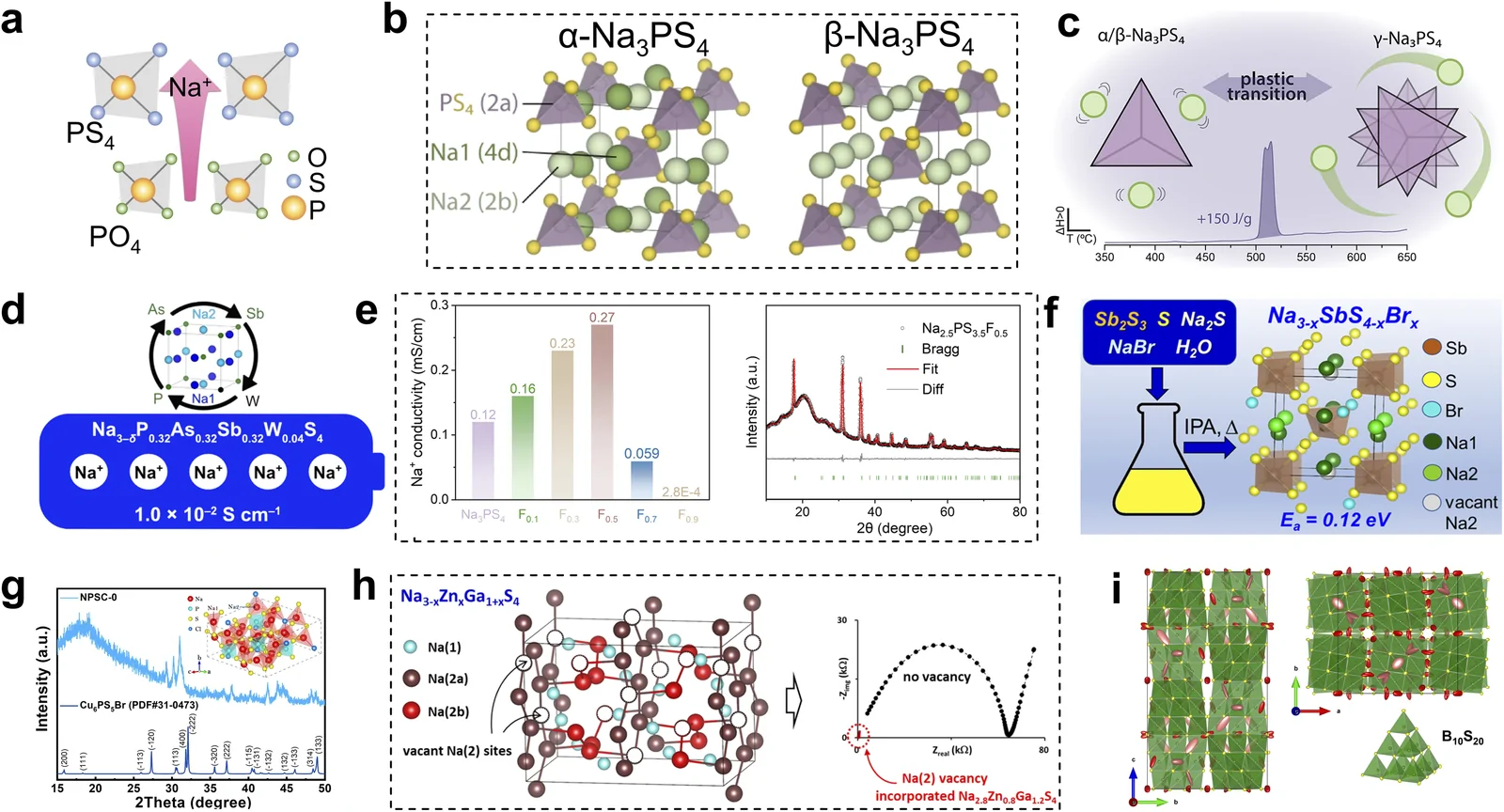
Structural and transport characteristics of representative sodium SEs. (a) Na^+^ transport channels in PS_4_ and PO_4_ frameworks. (b) Phase relationship of Na_3_PS_4_.^59^ Copyright 2021. *American Chemical Society*. (c) Plastic crystal transition of Na_3_PS_4_.^63^ Copyright 2019. *American Chemical Society*. (d) Structure of high entropy Na_3−*δ*_P_0.32_As_0.32_Sb_0.32_W_0.04_S_4_.^56^ Copyright 2025. *American Chemical Society*. (e) RT conductivity, Rietveld refinement, and 700 K Na^+^ probability densities of Na_3−*x*_PS_4−*x*_F_*x*_.^64^ Copyright 2025. The Royal Society of Chemistry. (f) Synthesis and structure of Na_3−*x*_SbS_4−*x*_Br_*x*_.^57^ Copyright 2021. *American Chemical Society*. (g) Structures of Na_6_PS_5_Cl.^65^ Copyright 2024 by the authors. Licensee MDPI. (h) Structures and impedance response of Na_3−*x*_Zn_*x*_Ga_1+*x*_S_4_.^66^ Copyright 2022. The Royal Society of Chemistry. (i) Structures of Na_3_B_5_S_9_.^67^ Copyright 2023. Wiley-VCH GmbH.

In the topological evolution of sulfide SEs, the polymorphic phase transition of Na_3_PS_4_ is a representative example for understanding the structure property relationship of ionic conduction. Its RT stable α-phase exhibits tetragonal symmetry (space group *P*4̄2_1_*c*). The relatively compact crystal framework constrains the Na^+^ migration channels, resulting in a RT impedance of only about 10^−6^ Ω cm^−1^.^62^ When the high temperature β-phase (body centered cubic, space group *I*4̄3*m*) is metastably retained at RT through a glass ceramic process the Na^+^ coordination environment changes from a low symmetry constrained configuration to a more isotropic three dimensional interconnected network, increasing the ionic conductivity to 10^−4^ S cm^−1^ (Fig. 2b).^23^ Temperature dependent Bragg diffraction, pair distribution function (PDF) analysis, Raman spectroscopy, and inelastic neutron scattering further revealed a third polymorph, γ-Na_3_PS_4_. In this phase, the PS_4_^3−^ anion sublattice exhibits liquid like rotational disorder in the solid state, providing additional degrees of freedom for Na^+^ diffusion and further enhancing ionic conductivity (Fig. 2c).^63^ On this basis, chemical substitution further expands the topological diversity of sodium sulfides. Zhang *et al.* introduced larger Sb^5+^ to replace P^5+^ in Na_3_PS_4_, which widened the ion migration bottleneck through lattice expansion and introduced intrinsic Na^+^ vacancies that activate efficient hopping, thereby increasing the RT conductivity of Na_3_SbS_4_ to the 10^−3^ S cm^−1^ level.^27^ A more complex example is Na_11_Sn_2_PS_12_. In this structure, PS_4_ and SnS_4_ polyhedra are closely connected through face sharing and edge sharing, forming a network of NaS_6_ octahedral sites with nearly degenerate site energies. This sublattice arrangement weakens the deep potential well confinement typical of conventional lattices, allowing Na^+^ carriers to undergo long range fast conduction through three dimensional isotropic channels with a low activation energy of approximately 0.25 eV and a RT ionic conductivity of up to 4 mS cm^−1^.^68,69^

Macroscopic ionic conductivity is governed by carrier concentration, charge, and ionic mobility. Accordingly, a key strategy for approaching the conductivity limit of sulfide electrolytes is to introduce defects that regulate carrier concentration and create structural disorder within an optimized transport framework. Heterovalent cation substitution is one of the most widely used methods for tuning such lattice defects. Through charge compensation, it can introduce interstitial Na^+^ or Na^+^ vacancies, disrupt ordered sublattice occupancy, and thereby optimize both carrier concentration and transport kinetics.^70^ In the Na_3_PS_4_ framework, partial substitution of P^5+^ by tetravalent cations, such as Si^4+^, Sn^4+^, or Ge^4+^, introduces additional interstitial Na^+^ and significantly increases carrier concentration.^71^

Recently, combining configurational entropy with heterovalent substitution has emerged as an effective strategy. By introducing configurational entropy through mixed Pn sites (P/As/Sb) and coupling it with W^6+^ substitution, Na_3−*δ*_P_0.32_As_0.32_Sb_0.32_W_0.04_S_4_ (N-PASS-W) achieved a RT conductivity of 10 mS cm^−1^ and a low activation energy of 0.15 eV (Fig. 2d). This result highlights the synergy between entropy driven disorder and heterovalent substitution, in which increased vacancy concentration and disordered site energies jointly lower the Na^+^ migration barrier.^56^ In addition, precursor purification further increased the conductivity of W doped Na_3−*x*_P_1−*x*_W_*x*_S_4_ to 26.4 mS cm^−1^.^30^ In Na_3_SbS_4_ electrolytes, the highest reported ionic conductivity has reached 32 mS cm^−1^.^26^

Anion defect engineering, including substitution of S^2−^ by Cl^−^, Br^−^, I^−^, or F^−^, can also induce Na^+^ vacancies and tune the electrostatic environment of the migration bottleneck through differences in anion size and polarizability. For example, Cl doped Na_3_PS_4_ exhibits a RT conductivity of up to 1.14 mS cm^−1^.^72^ F doped Na_2.5_PS_3.5_F_0.5_ introduces Na^+^ vacancies and activates coupled anion cation dynamics, giving a RT conductivity of 0.27 mS cm^−1^ and improved cold pressing density. *Ab initio* molecular dynamics (AIMD) predicts that its Na^+^ conductivity could reach 35.63 mS cm^−1^ at 300 K, providing a useful model for correlating defect chemistry with ion transport in sulfides (Fig. 2e).^64^ Br^−^ substitution in Na_3_SbS_4_ reduces the activation energy to approximately 0.1 to 0.12 eV by regulating bottleneck size (Fig. 2f).^57^ Jalem *et al.* further used density functional theory (DFT) and molecular dynamics (MD) calculations to show that Cl and Br doping lower the Na^+^ migration barrier in Na_3_SbS_4_ by modulating the bottleneck size between Na sites, with predicted activation energies as low as approximately 0.1 eV at 4% Na vacancy concentration.^73^

Systematic studies of Na_6_PS_5_X (X = Cl, Br, I) argyrodite type sulfides further revealed that halide ionic radius and polarizability can finely tune Na^+^ conduction, with conductivities exceeding 10^−4^ S cm^−1^ (Fig. 2g).^66,74^ Beyond the conventional Na_3_PS_4_ and Na_3_SbS_4_ frameworks, the chemical space of sodium sulfides continues to expand. Seo *et al.* partially substituted S^2−^ with I^−^ in Na_3_ZnGaS_4_, which perturbed the Na^+^ distribution, increased vacancy concentration, and shortened Na to Na distances, increasing ionic conductivity from 10^−3^ S cm^−1^ to 1.12 mS cm^−1^ (Fig. 2h).^75^ Zhou *et al.* reported the first sodium thioborate fast ion conductor, Na_3_B_5_S_9_, in which three dimensional Na^+^ diffusion channels constructed from B_10_S_20_ supertetrahedral clusters enabled a sintered body conductivity of 0.80 mS cm^−1^ and opened a new structural family for sodium sulfide SEs (Fig. 2i).^65^

However, static defect engineering and topological connectivity provide only the thermodynamic and geometric basis for high ionic conductivity. Further enhancement of macroscopic ion transport requires dynamic many body interactions within the disordered framework. In highly disordered, defect rich sulfide frameworks with high carrier concentrations, shortened Na^+^ to Na^+^ distances enhance coulombic interactions and promote correlated ion motion. He *et al.* revealed such multi-ion cooperative hopping in various fast-ion conductors using AIMD simulations, showing that it can reduce the apparent activation energy from 0.4 to 0.5 eV for isolated single ion hopping to 0.1 to 0.2 eV.^76^ In addition, low-frequency librational motions of PS_4_ or SbS_4_ polyhedra, often referred to as the paddle wheel effect, can couple with Na^+^ hopping in both space and time, transiently widening migration bottlenecks and further lowering the effective migration barrier.^77^

In summary, ultrafast Na^+^ transport in sulfide SEs is not governed by any single structural feature or chemical component. Instead, it arises from the coupled contributions of multiple physicochemical factors, including the flat potential energy landscape enabled by the high polarizability of S^2−^, highly symmetric and three dimensionally connected migration pathways, high concentrations of disordered defect states introduced by targeted doping, and many body cooperative hopping and phonon coupling driven by lattice dynamics. This combination of features gives sulfides their attractive transport advantages. However, the same electronic structural characteristics that enable rapid ion transport, particularly the high polarizability of S^2−^ and the high lying valence band, also constitute the primary source of their interfacial instability under electrochemical conditions.

### 2.2 Oxidative instability of sulfides at high voltage cathode interfaces

The exceptional Na^+^ conductivity of sodium sulfide SEs originates from the high polarizability and electron cloud flexibility of the S^2−^ anion. From a solid-state band structure perspective, however, higher anion polarizability is often associated with a higher valence band maximum (VBM). The high energy S 3p states that facilitate rapid Na^+^ transport also constrain the intrinsic oxidative stability of sulfides, with oxidation often occurring below approximately 2.5 V *vs.* Na^+^/Na.^47,78^ This limitation is not an accidental drawback of specific compositions, but reflects the shared electronic origin of the transport advantages and oxidative instability of sulfides. Although W doped Na_3−*x*_P_1−*x*_W_*x*_S_4_ has recently reached a conductivity of 26.4 mS cm^−1^ through Na_2_S precursor purification,^30^ and W doped Na_3_SbS_4_ systems have achieved conductivities above 30 mS cm^−1^,^26,56,58^ these compositional engineering strategies do not remove the intrinsic oxidative instability associated with the high VBM of S^2−^. Experiments further show that Br^−^ doping can increase conductivity and reduce activation energy, but leaves the thermodynamic electrochemical window nearly unchanged at approximately 1.83 to 1.90 V.^57^ Thus, the ultrafast ion conduction of sulfides and their narrow oxidation window are not independently tunable parameters. They instead represent the kinetic and thermodynamic consequences of the same S^2−^ based electronic structure. Bulk chemical modification of sulfides is therefore fundamentally a tradeoff between transport kinetics and thermodynamic stability.

Under high potential cathode conditions, this thermodynamic constraint leads to predictable interfacial decomposition. Taking Na_3_PS_4_ as an example, DFT calculations and quantitative X-ray Photoelectron Spectroscopy (XPS) analysis show that it tends to form elemental sulfur (S^0^), P_2_S_5_, and polysulfide species under oxidizing conditions (Fig. 3a).^48^ Phase-equilibrium calculations for solid–solid reactions between Na_3_PS_4_ and various cathode materials reveal similar degradation pathways. For oxide cathodes, O^2−^ can exchange with S^2−^ from PS_4_^3−^ to form PO_4_^3−^, providing a major thermodynamic driving force for cathode and sulfide interfacial reactions (Fig. 3b). For main group containing sulfides such as Na_3_SbS_4_, AIMD simulations show that close contact with layered cathodes such as NaCrO_2_ promotes sulfur migration toward the cathode side, triggering interfacial atomic rearrangement and structural degradation.^73^ Experimentally, ToF-SIMS and XPS analyses of composite cathodes detect clear SO_3_^2−^ and SO_4_^2−^ signals in the S 2p spectra (Fig. 3c), providing direct chemical evidence of oxidative decomposition at sodium sulfide cathode interfaces. Electrochemical impedance spectroscopy (EIS) and distribution of relaxation times (DRT) analyses further indicate that low and mid frequency impedance features arise from the coupled effects of chemical degradation and space charge depletion.^53^

**Fig. 3:**
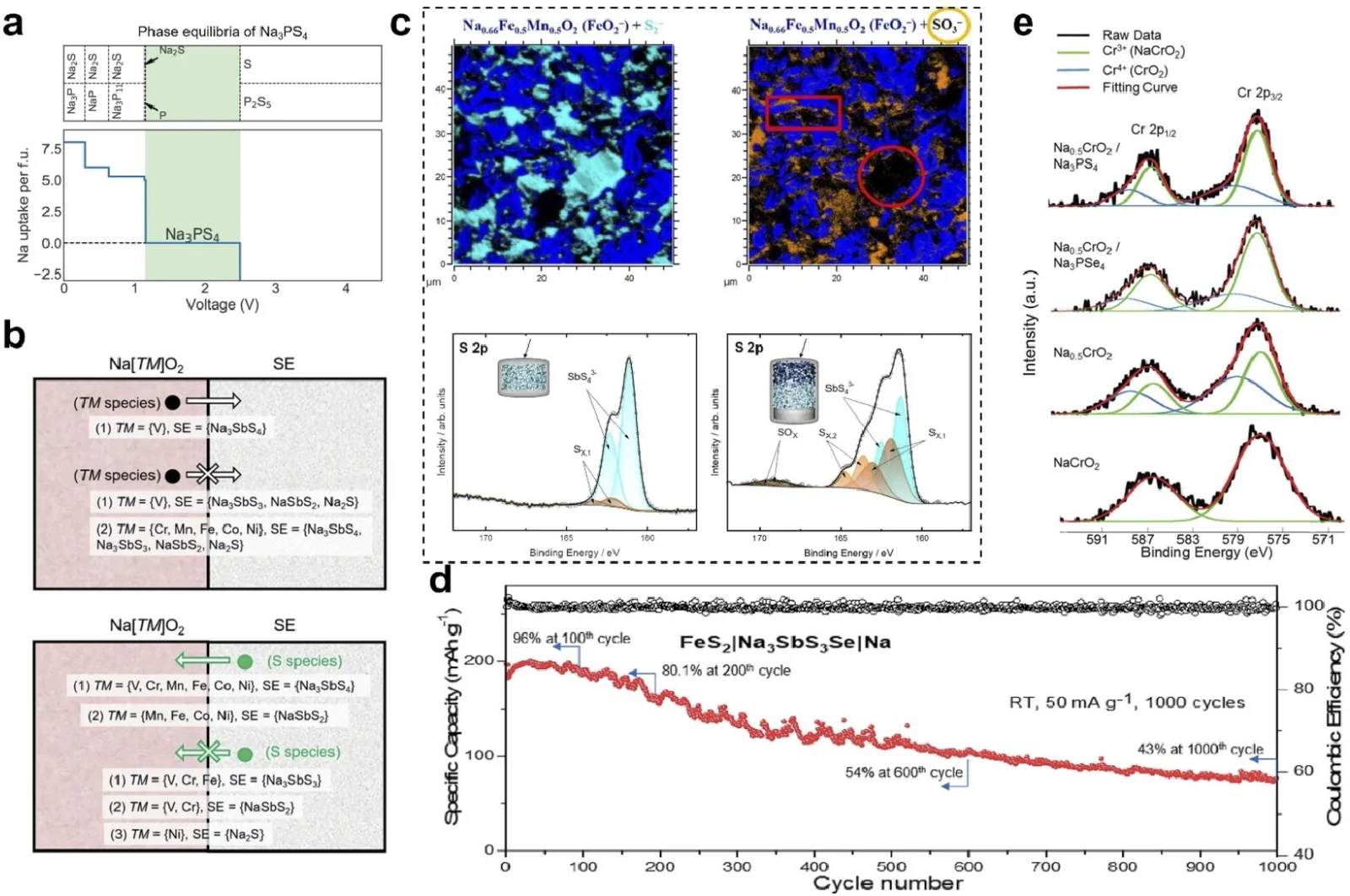
(a) Predicted phase equilibria and Na uptake of Na_3_PS_4_ as a function of voltage.^48^ Copyright 2017. *American Chemical Society*. (b) Schematic of transition metal (TM) and S migration across the Na[TM]O_2_ and SE interface.^73^ Copyright 2022. The Royal Society of Chemistry. (c) Time-of-flight secondary ion mass spectrometry (ToF-SIMS) and S 2p X-ray photoelectron spectroscopy (XPS) analyses of cycled sulfide-based cathode composites.^53^ Copyright 2024. *American Chemical Society*. (d) Cycling performance of a FeS_2_|Na_3_SbS_3_Se|Na cell at RT.^79^ Copyright 2023. Elsevier. (e) Cr 2p XPS spectra showing interfacial chemical evolution between Na_0.5_CrO_2_ and sulfide or selenide electrolytes.^47^ Copyright 2017. The Royal Society of Chemistry.

Low potential sulfur-based cathodes were once considered an engineering compromise to avoid sulfide oxidation, but carbon assisted catalytic decomposition also limits this strategy. Although sulfur cathodes offer high theoretical capacity and low cost, their poor electronic conductivity requires large amounts of conductive carbon in composite electrodes. High surface area carbon can act as active sites that catalyze sulfide oxidative decomposition during cycling. Na_3_SbS_3_Se prepared by a solvent free method enabled up to 1000 cycles in a Na‖FeS_2_ ASSSBs (Fig. 3d).^79^ However, oxidative decomposition of sodium thiophosphate in the Na_3_PS_4_/FeS_2_ system remains difficult to fully suppress. Thermal treatment can significantly alter the cathode interfacial chemistry and intensify the coupled degradation caused by carbon assisted interfacial catalysis and intrinsic sulfide decomposition.^80^ Even when a self-sacrificial interfacial strategy is used, in which Na_3_SbS_4_ decomposition is deliberately exploited so that it participates in multielectron redox reactions with elemental sulfur as a coactive material in Na and S batteries, an ultrahigh RT capacity exceeding 1976 mAh g^−1^ can be achieved.^50^ Nevertheless, despite the initially high capacity, long term reversibility is still limited by bulk chemical instability and stress concentration. Thus, the narrow oxidative stability window of sulfides not only restricts their direct pairing with high voltage oxide cathodes but also leaves them vulnerable to carbon assisted side reactions in low potential sulfur-based cathode systems.

Interface degradation on the cathode side goes beyond the scope of simple thermodynamic decomposition. High-voltage polarization profoundly induces elemental interdiffusion and chemical crosstalk between the oxide cathode and the sulfide electrolyte. Under conditions of deep desodiation, transition metal ions (such as Ni, Co, Mn, and Cr) in the layered oxide lattice undergo significant directed migration toward the electrolyte side, accompanied by the permeation of S and P framework elements toward the cathode side. XPS depth profiling precisely captured the cross-interface diffusion pathways of Cr ions at the NaCrO_2_–Na_3_PS_4_ solid–solid interface (Fig. 3e). More critically, such interfacial degradation products derived from elemental interdiffusion typically exhibit non-negligible electronic conductivity,^53^ thereby spontaneously forming a mixed ionic–electronic conductor (MIEC) interphase at the interface. The presence of this MIEC layer weakens the electronic insulation provided by the ideal passivation layer, providing a continuous electron transport pathway for the deep and sustained oxidative decomposition of the bulk SE.^47^ Even more critically, chemical degradation may also couple with localized physical fields. On the electrical scale, the enormous chemical potential difference between the oxide cathode and the sulfide drives the spatial redistribution of Na^+^ at the interface, inducing a broad space charge layer (SCL).^81^ The extreme depletion of localized sodium ions not only manifests macroscopically as a sharp increase in interfacial charge transfer impedance, but the strong localized built-in electric field it induces further weakens the thermodynamic barrier to oxidation of the sulfide at the microscopic dynamic level. On the mechanical front, the products of interfacial oxidation and interdiffusion (such as polysulfides and amorphous phases rich in transition metals) not only exhibit extremely poor ionic conductivity but also show severe mismatch in mechanical properties with the parent electrolyte phase.^78^ During long-term cycling, the superimposed layered oxides undergo a lattice volume “breathing effect” caused by deep sodium deintercalation.^82^ Under the long-term action of an alternating stress field, microcracks form in the interfacial phase and rapidly propagate, ultimately leading to irreversible delamination of the solid–solid point contacts, continuously exposing fresh electrolyte surfaces to a high-voltage oxidation environment. This chain-reaction feedback mechanism, which integrates cross-interface chemical interference, deterioration of localized electric field polarization, and microscopic mechanical pulverization and delamination constitutes the underlying physical logic behind the nonlinear increase in high-voltage cathode interface impedance in sulfide ASSSBs.

In summary, the oxidative degradation of sulfides on the cathode side is a systemic failure anchored by the intrinsic constraints of the S^2−^ electronic structure. It begins with the low oxidation potential caused by the high-energy 3p valence band of S^2−^, is amplified by the MIEC characteristics of decomposition products and carbon-induced catalysis, and is exacerbated by a positive feedback loop involving elemental interdiffusion, the space charge layer, and chemical-mechanical coupling. This analysis provides a clear performance boundary: relying solely on interface engineering or fine-tuning of composition typically makes it difficult to break through the ceiling of intrinsic oxidation stability while maintaining the ultra-high bulk conductivity of sulfides. This boundary clearly defines the performance limits of pure sulfide systems when paired with high-voltage oxide cathodes, thereby highlighting the engineering necessity of introducing halide SEs and constructing spatially decoupled double-layer heterostructures. However, while this asymmetric configuration successfully avoids oxidative degradation on the cathode side, it also fully exposes the sulfide to the Na metal anode, which exhibits extremely high thermodynamic reduction activity. Following two factors, which induce or suppress the nucleation and penetration of dendrites, directly determine the ultimate cycle life and safety threshold of the dual-layer system: (1) whether the sulfide framework can withstand the deep chemical/electrochemical reduction of sodium metal at extremely low potentials; (2) how interfacial byproducts will affect local ion transport and microscopic mechanical stress.

### 2.3 Reductive decomposition and passivation layer formation at Na metal anodes

If oxidative decomposition at high-voltage cathodes limits the energy density ceiling of sulfide SEs, reductive degradation at the Na metal anode (0 V *vs.* Na^+^/Na) is another key factor governing full cell cycle life. Compared with cathode side oxidation, the anode side reduction environment is more severe because the Fermi level of Na metal lies at very high energy, creating a strong thermodynamic driving force for electron injection into the electrolyte conduction band minimum (CBM) or other low lying unoccupied states. High throughput DFT calculations have shown that the reduction limits of sulfide SEs generally lie well above the Fermi level of alkali metal anodes. For example, the reduction decomposition potential of Na_3_PS_4_ is approximately 1.2 to 1.5 V *vs.* Na^+^/Na.^83^ Jalem *et al.* further predicted that Na_3_SbS_4_ also undergoes reductive decomposition at 0 V *vs.* Na^+^/Na, consistent with previous experimental observations (Fig. 4a).^73,84,85^

**Fig. 4:**
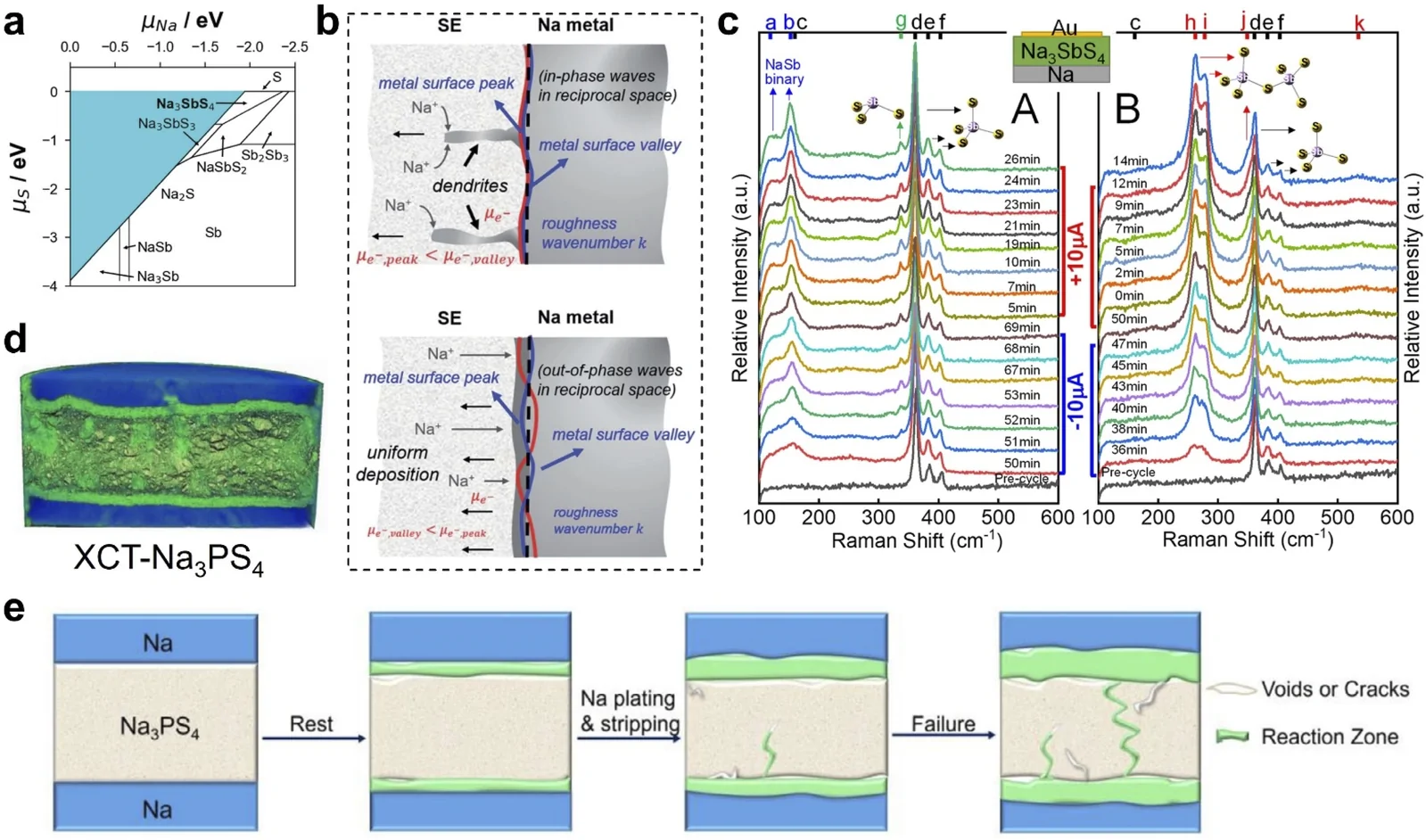
(a) DFT calculated thermodynamic stability window and (b) schematic of Na dendrite growth or stable deposition governed by electron chemical potential and surface roughness.^73^ Copyright 2022. The Royal Society of Chemistry. (c) *In situ* Raman evidence of interfacial decomposition.^15^ Copyright 2022. *American Chemical Society*. (d and e) Dynamic Na_3_PS_4_|Na failure involving reaction zone formation, voids or cracks, and electro chemo mechanical coupling.^49^ Copyright 2026. Wiley VCH.

Direct contact between Na_3_SbS_4_ and Na metal can generate Na_2_S and Na–Sb based decomposition products. Under typical electrodeposited surface roughness, the stress and electron density distributions at the Na_3_SbS_4_/Na interface further promote Na dendrite nucleation and growth. This indicates that, at the potential of Na metal, the surface and near surface regions of sulfide electrolytes are in a strongly nonequilibrium state, and electron injection from Na metal followed by reductive decomposition is thermodynamically favored (Fig. 4b).^73^ Electron injection therefore triggers a defined chemical decomposition pathway. For Na_3_PS_4_, the reduction reaction with Na follows the stoichiometry Na_3_PS_4_ + 8Na → Na_3_P + 4Na_2_S. XPS analysis provides direct evidence for this pathway, with the Na_2_S signal at 159.4 eV in the S 2p region and the Na_3_P signal at 124.8 eV in the P 2p region.^83^ The experimentally measured Na consumption ratio of approximately 1 : 8.4 is also in close agreement with the theoretical value.^49^ The reduction pathway of Na_3_SbS_4_ is more complex. *In situ* Raman spectroscopy revealed a multistep sequence in which SbS_4_^3−^ is gradually reduced to NaSb and Na_2_S through SbS_3_^3−^ and Sb_2_S_7_^4−^ intermediates (Fig. 4c).^15^ However, the ultimate interfacial behavior is governed not only by the decomposition reaction itself, but also by the electronic transport properties of the resulting products. This distinction represents a critical branching point that is often overlooked. When the interphase consists mainly of electronically insulating phases, such as NaF with a bandgap of approximately 11 eV or NaCl, a dense layer only a few nanometers thick can provide kinetic self-passivation because electron tunneling probability decays exponentially with thickness. In contrast, when the products include narrow bandgap phases such as Na_3_P with a bandgap of approximately 0.4 eV or Na_3_Sb with a bandgap of approximately 0.68 eV, or electronically conducting metallic species, an electron percolation network may form once these conductive products exceed the interfacial leakage threshold. The interface then shifts from kinetic self-passivation to a MIEC driven corrosion mode.^85^ Electrons can continuously penetrate into the electrolyte bulk along conductive pathways, expanding the reduction front from a two dimensional interface into three dimensional space. Microscopic electrical mechanism studies have shown that non self-limiting electronic conduction induced by localized reduction inside SEs can drive alkali metal nucleation and growth along grain boundaries and defects.^89^ The consequences of MIEC conduction extend beyond electronic degradation and can also drive mechanical failure. Alloying to form Na_15_Sn_4_ is associated with a theoretical volume expansion of approximately 420%, while formation of Na_3_Sb involves an expansion of approximately 290%. In a confined solid–solid contact region, the tensile and shear stresses generated by such large local volume mismatch can exceed the fracture resistance of the electrolyte matrix, initiating dense microcracks and driving their propagation. *In situ* X-ray computed tomography (XCT) imaging directly captured this coupled failure process. Within two hours of contact between Na_3_PS_4_ and Na, a reaction layer as thick as 65 µm had already formed. Volume expansion of decomposition products and internal electronic conduction form a coupled feedback loop that drives crack tips deeper into the electrolyte bulk (Fig. 4d).^49^ Fresh electrolyte surfaces exposed by cracking are immediately subjected to further reduction. Product expansion, crack propagation, re exposure, and repeated reduction together form a self-sustaining cycle that converts localized interfacial chemistry into bulk scale physical failure of the SE (Fig. 4e).

Collectively, sulfide degradation at the Na metal anode is not a simple superposition of independent failure modes, but a coupled causal chain rooted in electronic structure: (1) it begins with the mismatch between the electrolyte CBM or low-lying unoccupied states and the Na Fermi level; (2) its divergence is governed by the electronic transport properties of the decomposition products; (3) its amplification arises from positive feedback between MIEC leakage and chemomechanical damage; (4) its endpoint is physical collapse of the interface. This causal chain provides a clear engineering insight: composition tuning or microscopic encapsulation of sulfides alone is generally insufficient to resolve the intrinsic electronic structure conflict between high ionic conductivity and interfacial stability. In other words, the practical solution is not simply to make sulfides more stable against Na metal, but to prevent direct exposure of sulfides to metallic Na. This conclusion provides the scientific basis for constructing artificial interlayers that block electron injection, spatially separating sulfides from the lowest potential region, and introducing halide or oxide intermediate layers to improve anode interfacial compatibility. Representative sodium sulfide electrolytes are compared in Table 1, with emphasis on reported ionic conductivity, electrode compatibility, and actual cell configurations. The entries illustrate literature reported trends under different test conditions rather than a universal ranking of the materials.

**Table 1:** Comparison of representative sodium sulfide solid electrolytes in terms of ionic conductivity, electrochemical behavior, electrode compatibility, and mechanical characteristics. Some parameters were not reported in the cited studies and are marked as not reported (NR)

Electrolyte	*σ* (mS cm^−1^)	Cathode	Cutoff voltage	Anode	Cycling conditions	Capacity retention	Fabrication pressure	Ref.
**Thiophosphate and related sulfides**								
Na_3_PS_4_	0.73	NaNi_1/3_Fe_1/3_Mn_1/3_O_2_	2.3–4.2 V	Na|Na_3_Sb plus NaF layer	0.2C; 300th	88.3%	500–600 MPa	49
Na_3−*δ*_P_0.32_As_0.32_Sb_0.32_W_0.04_S_4_	10	NR	NR	NR	NR	NR	380 MPa	56
Na_2.8_P_0.8_W_0.2_S_4_	26.4, sintered; 15.5, cold pressed	NR	NR	NR	NR	NR	375 MPa	30
Na_2.9375_PS_3.9375_Cl_0.0625_	>1	TiS_2_	1.2 to 2.4 V	Na	C/10, 10th	80 mAh g^−1^ over 10th	360 MPa	72
Na_2.5_PS_3.5_F_0.5_	0.27	Na(Mn_1/4_Fe_1/4_–Co_1/4_Ni_1/4_)O_2_	1.8–4.1 V	Na_15_Sn_4_ + HC	2C, 1000th	88%	389 MPa	64
Na_5.5_PS_4.5_Cl_1.5_	1.2	Na_3_V_2_(PO_4_)_3_	1.4 to 1.8 V	Na	0.05C, 10th	73%	300 MPa	66
Na_3_PS_3.85_O_0.15_	0.27	S	1–3 V	Na	0.1 mA cm^−2^, 40th	80%	450 MPa	86

**Antimony based sulfides**								
Na_3_SbS_4_	0.1 to 0.2	FeS_2_	0.6 to 3.0 V	Na–Sn	50 mA cm^−2^, 50th	62%	380 MPa	87
Na_2.88_Sb_0.88_W_0.12_S_4_	32	NR	NR	NR	NR	NR	1080 MPa	26
Na_3−*δ*_Sb_0.8_(SnWCaTi)_0.05_S_4_	6.3	TiS_2_	1–2.5 V	Na_5_Sn	0.5C, 450th	75%	280 MPa	31
Na_2.85_SbS_3.85_Br_0.15_	∼2.87	TiS_2_	1.0 to 2.8 V	Na_15_Sn_4_	0.064 mA cm^−2^, 50th	48.9%	312 MPa	57
Na_3_SbS_3_Se	0.375	FeS_2_	1.0 to 2.7 V	Na	50 mA g^−1^, 1000th	43%	NR	79
Na_2.85_Sb_0.95_W_0.05_S_3.9_Cl_0.1_	12.66	TiS_2_	1–2.5 V	Na	0.1C, 100th	81.6%	180 MPa	58

**Other sulfide glasses and derivatives**								
Na_2.9_ZnGaS_3.9_I_0.1_	1.12	TiS_2_	0.8–2.5 V	Na_2_Sn	0.5C, 100th	89.7%	350 MPa	75
Na_3_BS_3_	0.011	Hard carbon	−0.15–2.5 V	Na–Sn	0.038 mA cm^−2^, 10th	80%	360 MPa	88

## 3. Halide solid electrolytes

Sulfides achieve fast ion transport through the highly polarizable S^2−^ framework, whereas halides follow a different design logic. The deep Cl^−^ valence band improves oxidative stability and cathode compatibility, but crystalline halides often require vacancy engineering, cation disorder, amorphization, or multi anion coordination to reach practical Na^+^ conductivity. This chapter summarizes the structural evolution, cathode side stability, and anode side reductive vulnerability of sodium halide SEs.

### 3.1 Structural evolution, vacancy regulation, and Na ion migration in sodium halides

At high-voltage cathode interfaces, to overcome the intrinsic thermodynamic instability of sulfide SEs caused by band mismatch, the design of solid-state battery materials has increasingly shifted toward anion frameworks with lower valence band maximum and highest occupied molecular orbital energy levels. Sodium halide SEs, especially chloride-based systems, have rapidly emerged as promising candidates for pairing with high energy density oxide cathodes because of their broad electrochemical stability windows, with theoretical oxidation potentials generally above 4.0 V *vs.* Na/Na^+^, and their favorable room temperature mechanical ductility.

From a crystal chemistry perspective, sodium halides include close packed Na_3_MCl_6_, Na_2_MCl_6_, and NaMCl_6_ families, UCl_3_ type halides, and Al-based halides. In the close packed families, M represents trivalent metals such as Y^3+^, Yb^3+^, and Er^3+^, tetravalent metals such as Zr^4+^ and Hf^4+^, or pentavalent metals such as Ta^5+^ and Nb^5+^. These materials are generally constructed from a nearly close packed Cl^−^ anion sublattice, in which Na^+^ and M cations occupy octahedral voids and form frameworks based on MCl_6_ octahedra. Depending on the stacking sequence of Cl^−^, the anion framework may adopt cubic close packed, hexagonal close packed, or mixed stacking arrangements (Fig. 5a and c).^90,91^ In contrast, UCl_3_ type halides are derived from a hexagonal high coordination framework. La based UCl_3_ type chlorides provide one dimensional Na^+^ migration channels along the crystallographic *c* direction. Zr substitution and mechanical activation can introduce Na vacancies and modify the channel geometry, as demonstrated by Na_0.7_La_0.7_Zr_0.3_Cl_4_.^90^ This result establishes UCl_3_ type halides as a complementary sodium halide family with transport characteristics distinct from those of the conventional close packed Na_3_MCl_6_ type materials. Al-based sodium halides form another distinct family because Al^3+^ preferentially adopts tetrahedral [AlCl_4_]^−^ or mixed Al/O/Cl coordination environments. Therefore, NaAlCl_4_ and related oxychlorides should not be regarded as direct members of the octahedral Na_3_MCl_6_ family.^93^

**Fig. 5:**
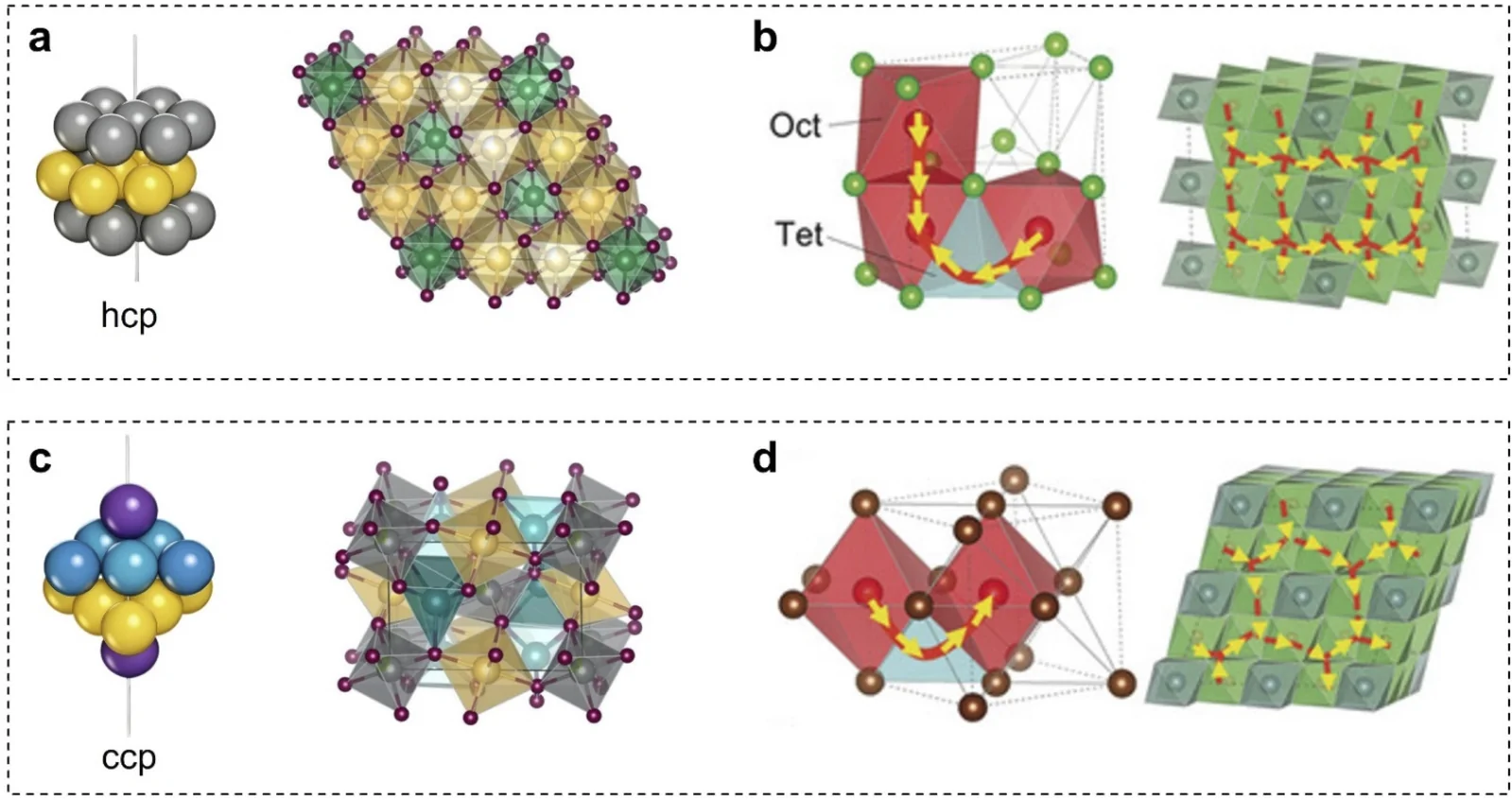
(a) Crystal structures of Na_2_ZrCl_6_ under hexagonal packing and (b) the corresponding ion transport pathways. (c) Crystal structures of Na_3_ErCl_6_ under cubic packing and (d) the corresponding ion transport pathways.^38,92^ Copyright 2020. *American Chemical Society*. Copyright 2019. Wiley VCH.

Compared with sulfide anions, chloride anions have higher electronegativity and lower polarizability.^16^ This more rigid anion framework improves oxidative stability but weakens the dynamic screening of migrating Na^+^ by the anion electron cloud. Consequently, Na^+^ experiences stronger electrostatic repulsion and a steeper potential energy landscape during migration. At the atomic scale, dense anion packing strongly governs the microscopic Na^+^ migration pathway. Between adjacent octahedral sites, Na^+^ cannot directly pass through shared polyhedral faces. Instead, it must migrate through a narrow tetrahedral site as a high energy transition state, following the classical octahedral tetrahedral octahedral pathway (Fig. 5b and d).^38,52^

Because the relatively rigid electron cloud of Cl^−^ cannot provide the flexible buffering effect found in sulfides, the size of the tetrahedral transition site becomes a critical electrostatic and geometric bottleneck for ion hopping. Strong electrostatic trapping therefore leads to sluggish Na^+^ transport in highly ordered structures. For example, in the RT stable phase of Na_3_YCl_6_, Na^+^ and Y^3+^ are highly ordered at octahedral sites, resulting in a Na^+^ migration barrier of approximately 0.8 eV and a RT ionic conductivity of only 10^−7^ S cm^−1^.^39,94^ Accordingly, constructing a continuous and low barrier Na^+^ migration network within dense halide lattices is the key scientific challenge for improving the ionic conductivity of sodium halides. To overcome the electrostatic trapping caused by ordered cation arrangements in halide crystals, the cation sublattice must be driven toward a more disordered and more symmetric state through thermodynamic or kinetic regulation. High energy ball milling has been shown to stabilize a monoclinic *P*2_1_/*n* Na_2_ZrCl_6_ polymorph with improved ionic conductivity, which is difficult to access through conventional solid-state reactions. In complex solid solution systems, such as Na_2.25_Y_0.25_Zr_0.75_Cl_6_, the degree of Y^3+^ and Zr^4+^ mixing at transition metal sites plays a decisive role in Na^+^ transport. Increased cation mixing partially activates Cl^−^ rotational motion (Fig. 6a),^17^ thereby facilitating long range Na^+^ migration. By linking synthesis route, polymorph formation, cation disorder, and ion transport, this strategy increases the ionic conductivity to 6.6 × 10^−5^ S cm^−1^ and enables stable operation for more than 1000 cycles in a practical NaCrO_2_ full cell.

**Fig. 6:**
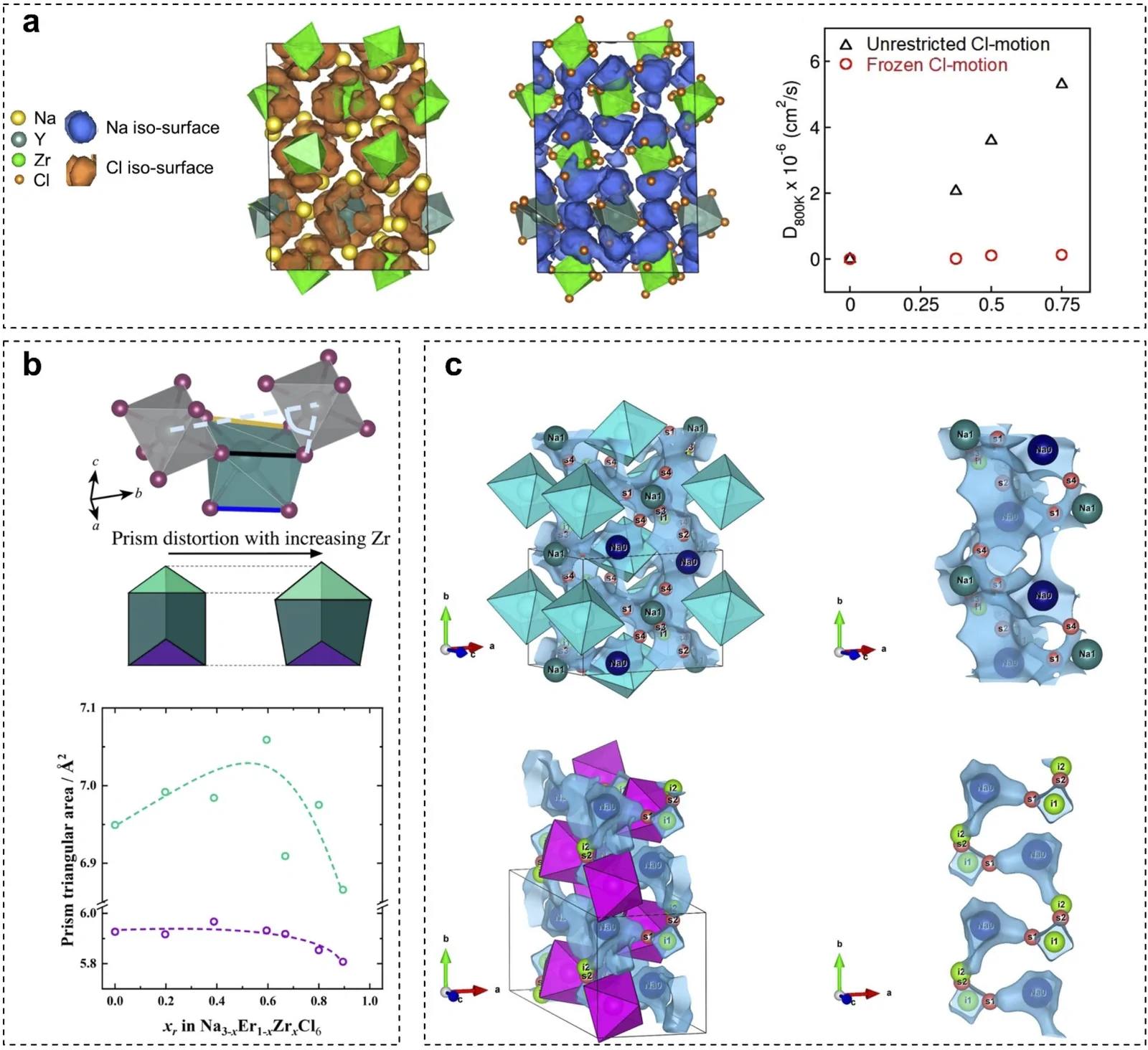
(a) AIMD probability density maps and Na^+^ diffusivity of Na_3−*x*_Y_1−*x*_Zr_*x*_Cl_6_ at 800 K.^17^ Copyright 2021. Springer Nature Limited. (b) Schematic of the distorted trigonal prismatic bottleneck for Na^+^ migration, where Zr induced octahedral rotation modulates channel geometry.^38^ Copyright 2020. *American Chemical Society*. (c) Bond valence energy landscapes of Na_3_YCl_6_ and NaNbCl_6_, showing that local polyhedral rotation and channel distortion generate more connected low energy Na^+^ migration pathways.^96^ Copyright 2026. The Royal Society of Chemistry.

On this basis, heterovalent cation substitution, in which M^3+^ is replaced by Zr^4+^, has become a central approach for introducing Na^+^ vacancies and activating diffusion pathways within the halide framework. In the Na_3−*x*_Er_1−*x*_Zr_*x*_Cl_6_ series, the composition with *x* = 0.6, namely Na_2.4_Er_0.4_Zr_0.6_Cl_6_, exhibits a sharp increase in ionic conductivity from 10^−9^ S cm^−1^ to approximately 0.04 mS cm^−1^. This enhancement is associated with local structural rearrangement of the polyhedral units induced by Zr^4+^ substitution (Fig. 6b).^38^ The material also shows better interfacial stability than Na_3_SbS_4_ in cathode composites, making this work one of the foundational studies on sodium ternary halide SEs.^53^ Na_2.5_Cr_0.5_Zr_0.5_Cl_6_, which adopts a cubic *Fm*3̄*m* structure, similarly forms a three dimensional isotropic diffusion network and achieves an ionic conductivity of approximately 0.1 mS cm^−1^.^95^

The effects of Nb^5+^ substitution on structure and transport kinetics have also been systematically investigated in the Na_3−2*x*_Y_1−*x*_Nb_*x*_Cl_6_ series. At *x* = 0.5, the conductivity increases from 10^−11^ S cm^−1^ to 10^−5^ S cm^−1^. Further increasing the Nb content, however, induces a structural threshold effect, where the distortion limit of Nb centered polyhedra restricts solid solubility (Fig. 6c).^96^ Al-based sodium halides provide a compositionally distinct route toward electrolytes based on earth abundant elements. Crystalline NaAlCl_4_ exhibits an ionic conductivity of approximately 3.9 × 10^−6^ S cm^−1^ at 30 °C. Partial replacement of Cl^−^ with F^−^ increases the conductivity to 3.56 × 10^−5^ S cm^−1^ for NaAlCl_3.3_F_0.7_ at 303 K, whereas BH_4_^−^ substitution produces only a limited improvement.^97^ In the Na_1+*x*_Zn_*x*_Al_1−*x*_Cl_4_ system, the Na_2_ZnCl_4_ type phase can accommodate approximately 34% Al^3+^ substitution and reaches 1.5 × 10^−5^ S cm^−1^ near the phase boundary.^98^ This composition should be regarded as a two phase system containing a limited Al substituted Na_2_ZnCl_4_ type phase rather than a continuous Zn substituted NaAlCl_4_ solid solution. In contrast, NaGa_*x*_Al_1−*x*_Cl_4_ forms a complete solid solution, but Ga incorporation progressively decreases the ionic conductivity. Although the NaGaCl_4_ end member exhibits a wider measured electrochemical stability range, its conductivity is only approximately 5 × 10^−7^ S cm^−1^ at 25 °C.^99^ Thus, cation and halogen substitution can regulate phase stability and electrochemical behavior, but does not fully overcome the slow Na^+^ transport of crystalline NaAlCl_4_.

Beyond conventional heterovalent substitution, intrinsic defect engineering offers a more targeted route for regulating ion transport. In mechanochemically synthesized nanocomposite electrolytes, DFT calculations combined with synchrotron X-ray and nuclear magnetic resonance (NMR) analyses have shown that interfacial oxygen substitution can markedly increase apparent ionic conductivity. Kwak *et al.* reported ZrO_2_, AX, and A_2_ZrX_6_ nanocomposite electrolytes, where A represents Li or Na and X represents Cl or F. By using Li_2_O or Na_2_O to generate a nanostructured network *in situ* during mechanochemical synthesis, the ionic conductivity of Na_2_ZrCl_6_ increased from 0.011 mS cm^−1^ to 0.11 mS cm^−1^ (Fig. 7a).^100^

**Fig. 7:**
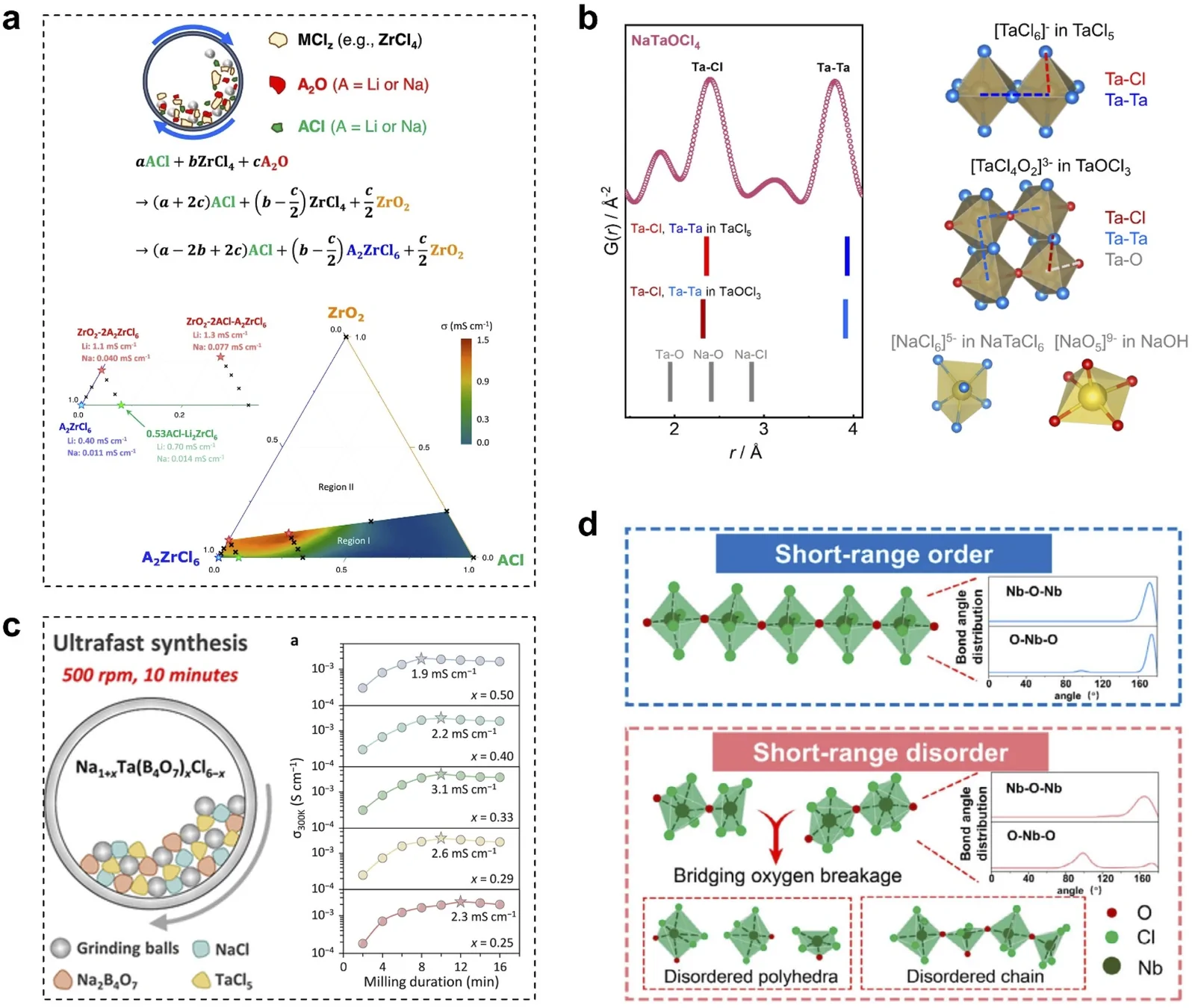
(a) One pot synthesis of ZrO_2_ containing A_2_ZrCl_6_ SEs and conductivity mapping at 30 °C.^17^ Copyright 2023. Springer Nature Limited. (b) Pair distribution function analysis of NaTaOCl_4_, showing local Ta–Cl, Ta–O, Na–Cl, and Na–O coordination motifs.^52^ Copyright 2024. *American Chemical Society*. (c) Rapid mechanochemical synthesis of Na_1+*x*_Ta(B_4_O_7_)_*x*_Cl_6−*x*_ and the corresponding conductivity evolution.^103^ Copyright 2026. *American Chemical Society*. (d) Schematic showing how short-range disorder promotes Na^+^ transport.^96^ Copyright 2026. Wiley VCH.

In NaTaCl_6_, Motohashi *et al.* first established its intrinsic sodium ion conducting behavior. Samples prepared by mechanochemical synthesis exhibited an ionic conductivity of 6.2 × 10^−5^ S cm^−1^ at 25 °C and an oxidation stability of 3.8 V *vs.* Na_15_Sn_4_.^101^ Subsequent studies further identified intrinsic Schottky defects, consisting of paired Na^+^ and Cl^−^ vacancies, as key contributors to ion transport. By precisely tuning Na and Cl stoichiometric defects, the ionic conductivity increased from 1.28 × 10^−5^ S cm^−1^ to 4.77 × 10^−4^ S cm^−1^, corresponding to an approximately 37-fold enhancement, without relying on amorphization. ^23^Na MAS NMR revealed that Schottky defects diversify local Na^+^ environments and thereby reduce the migration energy barrier. This work also showed that high- energy ball milling can simultaneously promote partial amorphization and induce uncontrolled defect formation, leading to conductivity variations of up to two orders of magnitude among samples prepared from the same batch.^102^ Despite substantial progress in cation and anion engineering within crystalline halide frameworks, the RT conductivity of most systems remains in the range of 10^−5^ to 10^−4^ S cm^−1^, which is still below the 10^−3^ S cm^−1^ level required for practical applications. In recent years, glassy and amorphous sodium oxychlorides, together with complex polyanionic halides, have become an important direction in the development of SEs. These materials offer crystal chemical strategies to mitigate the conflict between high ionic conductivity and electrostatic confinement imposed by hard anion frameworks. Recent studies have pushed the conductivity of sodium halide electrolytes to over 10^−3^ S cm^−1^.

One effective strategy is to construct asymmetric coordination environments by introducing dual anions with different electronegativities, ionic radii, and bonding preferences. In oxychloride systems, the coexistence of O^2−^ and Cl^−^ breaks the uniform coordination environment around high valent metal centers, distorts the MO_*x*_Cl_6−*x*_ polyhedra, and disrupts the periodic arrangement of Na^+^ sites. This local structural asymmetry weakens the rigid Na^+^ coordination environment, broadens the distribution of accessible migration bottlenecks, and creates a more continuous Na^+^ energy landscape. As a result, Na^+^ can migrate through a network of energetically similar local sites rather than being confined to fixed crystallographic positions. Amorphous oxychlorides NaMOCl_4_, where M represents Nb or Ta, achieved high Na^+^ conductivities of 1.2 to 1.5 mS cm^−1^, confirming the strong ion transport enhancement enabled by the mixed O and Cl anion framework.^52^ Zhou *et al.* further reported a scalable synthesis of NaTaOCl_4_, achieving a conductivity of 1.2 mS cm^−1^ through simple mixing followed by annealing at 100 °C, demonstrating the practical potential of oxychloride electrolytes (Fig. 7b).^104^

More complex polyanionic architectures further enhance Na^+^ transport. The Na_1+*x*_Ta_4_(B_4_O_7_)_*x*_Cl_6−*x*_ halide borate mixed anion system achieved a Na^+^ conductivity of 3.1 mS cm^−1^ after only 10 min of ball milling (Fig. 7c). AIMD simulations showed that borate incorporation weakens Na^+^ coordination and activates coupled cation and anion dynamics.^103^ In the carbonate containing oxychloride system derived from TaCl_5_ and *x*Na_2_CO_3_, the optimized TaCl_5_–0.5Na_2_CO_3_ (TC–NCO) composition further increased the ionic conductivity to 5.01 mS cm^−1^, highlighting the advantage of the carbonate route for rapid preparation of high conductivity electrolytes.^43^

Glassy network design provides another route to high conductivity. Superionic glasses with dual anion sublattices, such as 0.5Na_2_O–TaCl_5_ (NTOC), generate highly disordered Na^+^ distributions and low migration barriers through local mismatch between bridging and nonbridging oxygen atoms, resulting in a RT conductivity of 4.62 mS cm^−1^. This glass electrolyte also shows excellent cold pressing formability. Ojelade *et al.* used Na_2_O as a glass modifier to amorphize NaTaCl_6_, achieving an ionic conductivity of 4.41 mS cm^−1^ and a low electronic conductivity of 6.72 × 10^−10^ S cm^−1^ after only 4 h of ball milling. This result supports the use of inexpensive glass modifiers for low cost preparation of high conductivity glassy electrolytes.^105^

Nanocomposite design can further combine fast-ion transport with mechanical reinforcement.^14^ By dispersing NaCl and Ta_2_O_5_ nanograins throughout an amorphous oxychloride matrix, an ionic conductivity of 2.5 × 10^−3^ S cm^−1^ was achieved at 25 °C, together with an electrochemical window of 0.4 to 4.1 V *vs.* Na^+^/Na and improved macroscopic shear strength of the electrolyte separator.^106^ At the processing level, rapid quenching provides a powerful means of controlling both long range and short range disorder. Ultrafast cooling freezes disrupted M–Cl coordination and induces asymmetric M–O bonding, leading to polyhedral distortion and network connectivity disruption. As a result, NaNbOCl_4_ reaches an ionic conductivity of 1.51 mS cm^−1^, while quenched NaTaOCl_4_ reaches 7.2 mS cm^−1^, representing a record high value among reported sodium halide electrolytes (Fig. 7d).^42^ A similar strategy has been extended to Al-based electrolytes, in which oxygen incorporation and structural disorder effectively enhance Na^+^ transport. The viscoelastic glass NaAlCl_2.5_O_0.75_ reaches an ionic conductivity of 1.33 × 10^−3^ S cm^−1^ at 30 °C with a Na^+^ transference number of 0.91.^107^ A self propagating reaction involving sodium halide salts, AlCl_3_, and Sb_2_O_3_ produces amorphous Al-based oxyhalides containing NaCl, NaBr, or NaI, with conductivities generally in the range of 10^−4^ to 10^−3^ S cm^−1^.^108^ Reaction between NaAlCl_4_ and Na_2_O produces a composite containing NaAlCl_4_, Al_2_O_3_ nanoparticles, and interfacial oxychloride species. The optimized CSE0.75 composition reaches 1.3 × 10^−4^ S cm^−1^ at 30 °C, but the conductivity enhancement originates from the composite interfaces rather than from a homogeneous NaAlCl_4−2*x*_O_*x*_ solid solution.^93^ Amorphous AlCl_3_/0.75Na_2_O similarly reaches 1.70 × 10^−4^ S cm^−1^ at 30 °C, with pair distribution function analysis, solid state NMR, and simulations supporting the formation of Al/O/Al bridging environments.^109^ More recently, the dual oxygen precursor glass 0.6NaClO/AlCl_3_/0.175SeO_2_ achieved an ionic conductivity of 2.03 × 10^−3^ S cm^−1^ and a Na^+^ transference number of 0.87.^110^ These results demonstrate that mixed O/Cl coordination, amorphization, and interfacial oxychloride formation can substantially improve Na^+^ transport in Al-based electrolytes.

A different Al-based design uses mesoporous AlF_3_ to construct defective NaCl/AlF_3_/NaF heterointerfaces. The NH54 composition reaches 6.54 × 10^−5^ S cm^−1^ at 30 °C, while increasing the NaCl fraction further raises the conductivity above 10^−4^ S cm^−1^. The enhanced transport is associated with defective NaCl domains and low barrier NaCl/AlF_3_ and NaCl/NaF interfaces rather than a single crystalline phase. NH54 also retains measurable conductivity after exposure to 35% relative humidity, indicating better processing tolerance than conventional moisture sensitive chloride electrolytes. Nevertheless, its conductivity remains below that of the best Al-based oxychloride glasses.^111^

Meanwhile, multi-anion design is expanding into broader compositional spaces. Kalyk, Zeier, and colleagues extended this strategy from O/Cl to S/Cl systems. The incorporation of S^2−^ into an amorphous Ta^5+^ halide framework produced structural units distinct from those found in oxychlorides, and the Na_1.2_TaCl_3.8_S_1.2_ composition achieved a RT ionic conductivity of 3.8 mS cm^−1^.^112^ In crystalline Na_2_ZrCl_6_, site selective S^2−^ incorporation generated the cubic superionic conductor Na_3_ZrCl_5_S through vacancy coupled substitution. Neutron diffraction refinement confirmed that S^2−^ occupies 16.7% of the anion sites and introduces Na^+^ vacancies, yielding an ionic conductivity of 0.753 mS cm^−1^, approximately 40 times higher than that of Na_2_ZrCl_6_. When paired with a NaCrO_2_ cathode in an ASSSBs, this electrolyte sustained more than 1000 cycles at 0.3C with 83% capacity retention.^113^

These findings show that the anion chemistry of halide electrolytes is no longer restricted to a single Cl^−^ framework or simple O/Cl regulation. Local coordination environments, defect concentrations, and Na^+^ migration pathways can instead be systematically tuned through the synergistic incorporation of S^2−^, F^−^, O^2−^, and polyatomic anions. The concept of solid-state dissociation further broadens the design space for sodium halides. In this strategy, halide or oxychloride van der Waals frameworks act as solid state solvents that dissociate different metal salts, allowing electrolyte composition to be regulated in a manner analogous to liquid electrolyte formulation.^114^ This approach suggests that future sodium halide electrolyte design may move beyond conventional doping and substitution toward multi salt synergy based on solid state solvation and high throughput compositional screening. Overall, these studies mark a transition from single Cl^−^ framework design to multi anion synergy, with the compositional space extending from simple anion combinations such as O/Cl, S/Cl, and F/Cl to BO_*x*_/Cl, CO_3_/Cl, and more complex solid solvation systems.

In summary, sodium halide SEs have evolved from heterovalent defect induced vacancy restructuring in crystalline halides, to ion transport activation in oxychlorides through dual anion asymmetric coordination and amorphization, and further to rapid quenching in polyanionic systems. This evolution represents a shift from ordered lattices with severe ion transport limitations to disordered multiscale network topologies. Over this course, RT ionic conductivity has increased by several orders of magnitude, from early values near 10^−9^ S cm^−1^ or even insulating behavior, to the level of 10^−5^ S cm^−1^, and finally to 7.2 mS cm^−1^ in quenched NaTaOCl_4_. These advances have substantially revised the long-standing perception that hard anion systems inherently suffer from low RT conductivity, providing a kinetic foundation for exploiting their high oxidative stability. However, consistent with the fundamental tradeoff predicted by solid-state band theory, the wide high-voltage stability window of halides is accompanied by pronounced reduction instability at low-potential anode interfaces. This full cell compatibility paradox will be examined in the following sections.

### 3.2 Electronic origins of cathode interfacial compatibility in halide electrolytes

Crystal topology and vacancy network engineering can partly mitigate the kinetic limitations of halide SEs. However, the key reason halides can be coupled directly with oxide cathodes operating at 4.0 V or higher is their intrinsic thermodynamic resistance to oxidation. Within the thermodynamic framework of solid-state battery interfaces, the high-voltage oxidative stability of an electrolyte is closely related to the absolute energy level of its VBM.^78^ High-throughput DFT calculations have confirmed a quantitative relationship between electronic structure and oxidation stability. Qie *et al.* systematically evaluated the oxidation resistance of sodium halides at the first principles level, reporting electrochemical stability windows of 0.51 to 3.75 V for Na_3_YCl_6_ and 0.57 to 3.36 V for Na_3_YBr_6_. Their oxidation limits exceed 3.3 V, much higher than typical sulfide values below 2.5 V (Fig. 8a). Density of states (DOS) analysis further revealed the electronic origin of this redox advantage. In Na_3_YCl_6_, the VBM is dominated by Cl 3p nonbonding states, whereas the CBM mainly derives from empty Y 4d orbitals.^94^ The high electronegativity of Cl^−^ (Pauling electronegativity 3.16, compared with 2.58 for S^2−^) lowers the Cl 3p nonbonding states relative to S 3p states, thereby increasing the energy required for electron extraction during oxidation.^115^

**Fig. 8:**
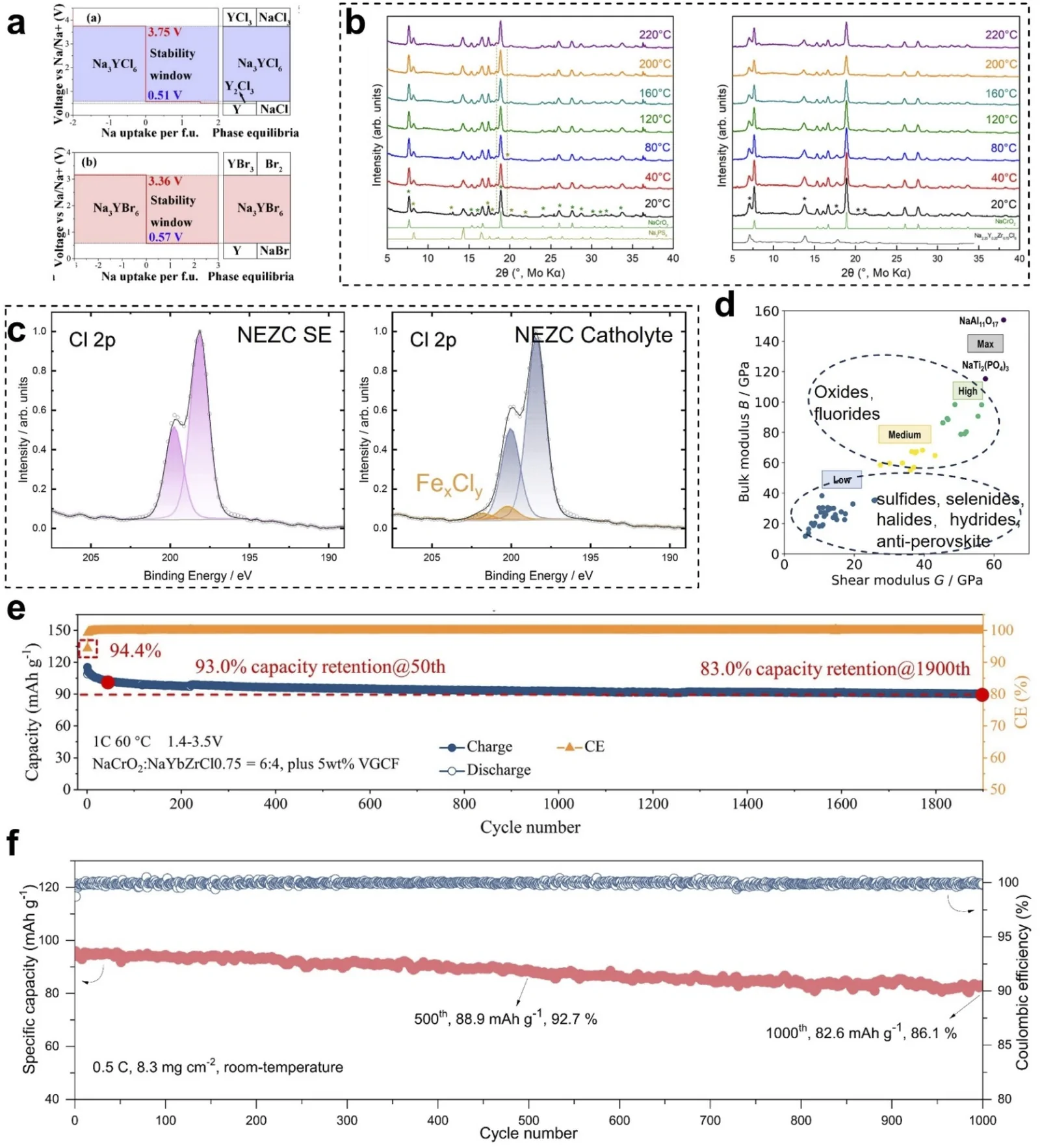
(a) Voltage profiles and phase equilibria of Na_3_YCl_6_ and Na_3_YBr_6_ during sodiation and desodiation.^94^ Copyright 2020. *American Chemical Society*. (b) Phase compatibility of Na_3_PS_4_ or Na_2.25_Y_0.25_Zr_0.75_Cl_6_ (NYZC0.75) with NaCrO_2_, showing that Na_3_PS_4_ degradation mainly arises from high voltage oxidation.^17^ Copyright 2021. Springer Nature Limited. (c) Cl 2p XPS evidence of interfacial changes in Na_2.4_Er_0.4_Zr_0.6_Cl_6_ (NEZC) composite cathodes.^53^ Copyright 2024. *American Chemical Society*. (d) K means clustering of Na ion conductors by bulk and shear moduli.^46^ Copyright 2025. The Royal Society of Chemistry. (e) Cycling performance of NYZC0.75.^82^ Copyright 2023. Elsevier. (f) Amorphous Na_1.4_Zr_0.4_Ta_0.6_Cl_6_ (NTZC) based ASSSBs.^117^ Copyright 2025. *American Chemical Society*.

A systematic DFT screening of 26 Na_3_MCl_6_ derivatives by Park *et al.* further confirmed the generality of this trend. Chlorides based on Group 3 and lanthanide elements generally exhibit oxidation limits above 3.5 V, whereas those based on p block elements and 3d transition metals show lower oxidation limits.^116^ Thus, the oxidation limit of Na_3_YCl_6_ (3.75 V) should not be viewed as the upper bound for sodium halides, but as the DFT calculated value for this specific composition in the *P*2_1_/*n* structure. Moving toward higher valent central metals, such as Zr^4+^ or Ta^5+^, or more electronegative anions, such as F^−^, can further increase the oxidation limit. Overall, a deep VBM and strong M and Cl bonding constitute the common chemical basis for the high oxidative stability of halide SEs.^91^

Another key advantage of halide cathode interfaces is their relative chemical inertness toward transition metal interdiffusion. In deeply desodiated layered oxide cathodes, high-valent transition metal ions, such as Cr^3+^/Cr^4+^, Ni^3+^/Ni^4+^, and Mn^4+^, are strongly oxidizing. For these ions to migrate across the interface into a halide lattice, they must overcome the kinetic barrier imposed by the dense Cl^−^ framework. Wu *et al.* directly supported this point using temperature programmed X-ray diffraction (XRD). No new diffraction peaks were observed in the Na_2.25_Y_0.25_Zr_0.75_Cl_6_ (NYZC0.75) and NaCrO_2_ powder mixture between 20 and 220 °C (Fig. 8b).^17^ Goodwin *et al.* further showed by ToF-SIMS and XPS depth profiling that, after several hundred cycles, only trace Fe_*x*_Cl_*y*_ species were detected at the halide cathode interface, whereas the sulfide cathode interface was heavily covered by oxidized products such as SO_3_^−^ and polysulfides (Fig. 8c).^53^

Compared with sulfide SEs, the space charge layer (SCL) effect driven by electrochemical potential differences at halide and cathode interfaces is also weaker. The deeper VBM of halides reduces the chemical potential mismatch with the cathode, thereby weakening the thermodynamic driving force for interfacial Na^+^ depletion.^81,91^ DRT analysis provides indirect experimental support: the interfacial peak positions in halide composite cathodes remain relatively stable during long term cycling, whereas the corresponding peaks in sulfide SEs continue to deteriorate.^53^ Together, resistance to chemical interdiffusion and weaker SCL formation shift the dominant failure modes at halide cathode interfaces from deep chemical decomposition and charge depletion, as seen in sulfides, toward slower physical contact degradation and mechanical accommodation during extended cycling.

From a mechanical perspective, an important advantage of halides is their low elastic modulus and favorable plastic deformability. Torii *et al.* systematically calculated the elastic properties of approximately 40 sodium-ion conductors using DFT and the Voigt Reuss Hill approximation, establishing a quantitative elastic modulus database for sodium solid-state electrolytes. Clearly, this study revealed that chlorides and sulfides generally exhibit low elastic moduli and high Pugh ratios (B/G > 1.75), indicating a tendency toward plastic deformation, whereas oxides are significantly stiffer (Fig. 8d). Specifically, the calculated Young's moduli are 15.31 GPa for monoclinic NaTaCl_6_, 21.65 GPa for trigonal Na_2_ZrCl_6_, 17.68 GPa for orthorhombic NaAlCl_4_, and 29.57 GPa for monoclinic Na_3_ErCl_6_.^46^ Nanoindentation measurements agree well with these calculations. Li, Xia, and coworkers measured the Young's modulus of NaTaCl_6_ to be 16.05 GPa.^44^ Shi *et al.* also noted that typical sulfides and halides have Young's moduli above 10 GPa.^45^ By contrast, Na_3_Zr_2_Si_2_PO_12_ (NASICON) type oxide SEs typically show Young's moduli as high as 100 to 150 GPa, making halides much softer than oxide ceramics. This low modulus allows halides to form conformal contact with cathode particles under mild cold-pressing and to relax stress concentrations through limited plastic flow under cyclic stress, thereby suppressing the initiation and propagation of interfacial microcracks.

The oxidative stability provided by a deep VBM, the resistance to chemical interdiffusion enabled by a dense Cl^−^ framework, and the mechanical adaptability arising from a low elastic modulus together give halide electrolytes one of their most attractive engineering features: the possibility of direct integration with bare cathodes. In contrast to sulfide systems that often require cathode surface coatings or buffer layers, unmodified layered oxide or polyanionic cathodes can show low interfacial impedance and high reversible capacity after direct cold-pressed contact with halides. This behavior has been verified in multiple independent cathode and halide combinations. NaCrO_2_ paired with NYZC0.75 achieved a first cycle efficiency of 97.1% and retained 89.3% capacity after more than 1000 cycles at 40 °C and 1C.^17^ NaCrO_2_ paired with NYZC0.75 retained 83.0% capacity after more than 1900 cycles at 60 °C and 1C (Fig. 8e).^82^ In Na_0.4_Ta_0.236_La_0.472_Cl_3_, NaCrO_2_ retained 89.7% capacity after 300 cycles at room temperature and 0.3C.^118^ NaCrO_2_ retained 82.9% capacity after 500 cycles at 60 °C and 1C in low cost NaAlCl_4_,^119^ demonstrating that even crystalline NaAlCl_4_ can function as a cathode side electrolyte despite its relatively low room temperature conductivity. In recent studies on Al-based oxychlorides, reported full-cells have demonstrated the feasibility of spatially separated electrolyte designs. NaAlCl_2.5_O_0.75_ enabled an Na_3_(VOPO_4_)_2_F (NVOPF) cell to retain 83.5% of its capacity after 600 cycles at 60 °C and 1C.^107^ An AOC/2NaCl catholyte delivered an initial reversible capacity of 106.2 mAh g^−1^ and retained 83.5% of this capacity after 60 cycles at 25 °C and 0.1C.^108^ The dual oxygen precursor NACDO glass enabled an uncoated NaNi_0.4_Fe_0.2_Mn_0.4_O_2_ cathode to retain more than 80% of its initial capacity after 480 cycles at 40 °C and 0.3C.^110^ These results support the use of Al-based oxychlorides as catholytes for oxide cathodes. However, because these cells employed additional electrolyte layers and alloy anodes, they should not be interpreted as evidence of direct stability against Na metal. Na_0.95_Ni_0.33_Fe_0.33_Mn_0.33_O_2_ (NNFMO) paired with fully amorphous Na_1.4_Zr_0.4_Ta_0.6_Cl_6_ achieved a high cathode loading of 20.85 mg cm^−2^ and retained 77.45% capacity after 450 cycles at 0.5C.^44^ Na_3_V_2_(PO_4_)_3_ paired with an amorphous coordination disordered chloride retained 86.1% capacity after 1000 cycles at 0.5C (Fig. 8f).^117^ These results span different halide compositions, cathode chemistries, and cycling conditions, collectively showing that the cathode side advantage of halides arises primarily from their intrinsic material properties rather than from additional interfacial modifications.

Based on the above analysis, cathode side compatibility in sodium halide SEs is not governed by a single physical property, but by the synergy of multiple intrinsic attributes: a deep VBM provides oxidative stability, a dense Cl^−^ framework suppresses chemical interdiffusion, a low elastic modulus improves mechanical compatibility, and a weaker SCL effect reduces interfacial charge depletion. From thermodynamic barriers to bare cathode integration, research on halide cathode interfaces has progressed from qualitative comparison to quantitative validation. However, the electrochemical window of inorganic SEs is ultimately constrained by orbital hybridization and the energy levels of both anions and cations. The electronegative anions, such as Cl^−^ or F^−^, and high valent central metals, such as Zr^4+^ or Ta^5+^, that give halides strong oxidative stability also introduce a corresponding drawback. Their empty metal d orbitals often dominate the shallow CBM, making halides susceptible to electron injection at low potentials near the Na metal anode and leading to non-self-limiting reduction. This tradeoff between oxidation resistance and reduction resistance is discussed in the next section.

### 3.3 Anode side reduction instability of halide electrolytes

Although sodium halide SEs exhibit excellent oxidative stability on the cathode side, their behavior toward low potential anodes remains a critical limitation. Direct reduction evidence for most Na_3_MX_6_ type halides has been obtained primarily at sodium metal and alloy interfaces, where electron injection and reduction of the halide framework can produce resistive or mixed conducting interphases. Hard carbon should be assessed separately because it does not form a single metal electrolyte boundary. Instead, its porous and electronically conductive structure creates distributed contact regions with the electrolyte. Accessible pores, defects, oxygen containing groups, and low potential sodium storage sites may therefore promote electrolyte reduction and irreversible sodium consumption throughout the composite electrode. At present, direct chemical and electrochemical evidence for completely dry interfaces between hard carbon and pure sodium halide electrolytes remains limited. Hard carbon compatibility should therefore not be inferred solely from either sodium metal data or cathode side halide stability. Systematic evaluations of cathode composite materials have shown that, for both chlorides and amorphous oxychlorides, extending their application from the cathode side to the anode side leads to severe reductive decomposition, which becomes a major intrinsic cause of full cell failure.^51,52^ Even with relatively high-potential alloy anodes such as Na_15_Sn_4_, halides can still exhibit thermodynamic instability beyond the tolerance of practical full cells.^120^ These findings indicate that halides are strongly prone to reductive decomposition near the potential of metallic sodium.

In practical evaluations of ASSSBs, Na_2.4_Er_0.4_Zr_0.6_Cl_6_ has been confirmed as a suitable cathode side electrolyte, but it suffers from severe reductive instability on the anode side.^53^ Deysher *et al.* compared Na_2.25_Y_0.25_Zr_0.75_Cl_6_, Na_3_PS_4_, and Na_2_(B_10_H_10_)_0.5_(B_12_H_12_)_0.5_ against Sn based alloy anodes using full cells and Na_9_Sn_4_|SE|Sn half cells.^51^ After cycling, Energy Dispersive Spectroscopy (EDS) mapping revealed a thick Na rich interphase of about 90 µm at the chloride and Sn interface, compared with about 10 µm for Na_3_PS_4_ and no detectable interphase for the borohydride. EIS showed severe interfacial resistance growth for the chloride electrolyte, while X-ray photoelectron spectroscopy (XPS) and X-ray diffraction (XRD) confirmed reduction of Zr and Y to metallic species together with NaCl formation. Direct current (DC) polarization further indicated that the reduced chloride interphase became electronically conductive, enabling continuous interphase growth and sodium inventory loss (Fig. 9a).^51^ Zhao *et al.* similarly showed that NaMOCl_4_, where M is Nb or Ta, is unstable against metallic Na, based on time dependent impedance growth and increasing overpotential in Na|NaMOCl_4_|Na cells (Fig. 9b).^52^ These results indicate that low lying conduction bands and reducible high valence metal centers make many sodium halides intrinsically vulnerable at low potential anodes. However, direct sodium specific studies remain limited, especially for *operando* interphase chemistry, depth resolved composition, electron transport, and mechanical failure, which represents a key gap for future research. Al-based chlorides and oxychlorides show the same general reduction limitation. The NaAlCl_4_/Al_2_O_3_/oxychloride composite begins to decompose at approximately 1.25 V *versus* Na_3_Sn and exhibits rapid polarization growth when placed directly against a Na_3_Sn electrode.^93^ NACDO also shows reductive decomposition beginning at approximately 2.2 V in electrochemical measurements.^110^ Accordingly, the reported NaAlCl_2.5_O_0.75_ cell used NASICON to prevent direct contact with Na metal, the AOC/2NaCl cell used a Na_2.9_PS_3.9_Cl_0.1_ separator and a Na/Sn alloy anode, and the NACDO cell used a Na_3_PS_4_ interlayer and a Na_15_Sn_4_ anode.^107^ These configurations confirm cathode side functionality but do not demonstrate direct compatibility between Al-based oxychlorides and Na metal. The NaCl/mesoporous AlF_3_ heterostructure provides more direct sodium plating and stripping evidence. Na/NH54/Na symmetric cells operated for more than 1000 h at 30 °C or 60 °C under low current densities.^111^ Post cycling analysis indicates the formation of a F and Cl containing interphase on Na. However, a small amount of liquid electrolyte was introduced as an interfacial wetting agent to improve physical contact. The result therefore demonstrates a promising wetting assisted Na interface rather than a completely dry and intrinsically stable NH54/Na interface.

**Fig. 9:**
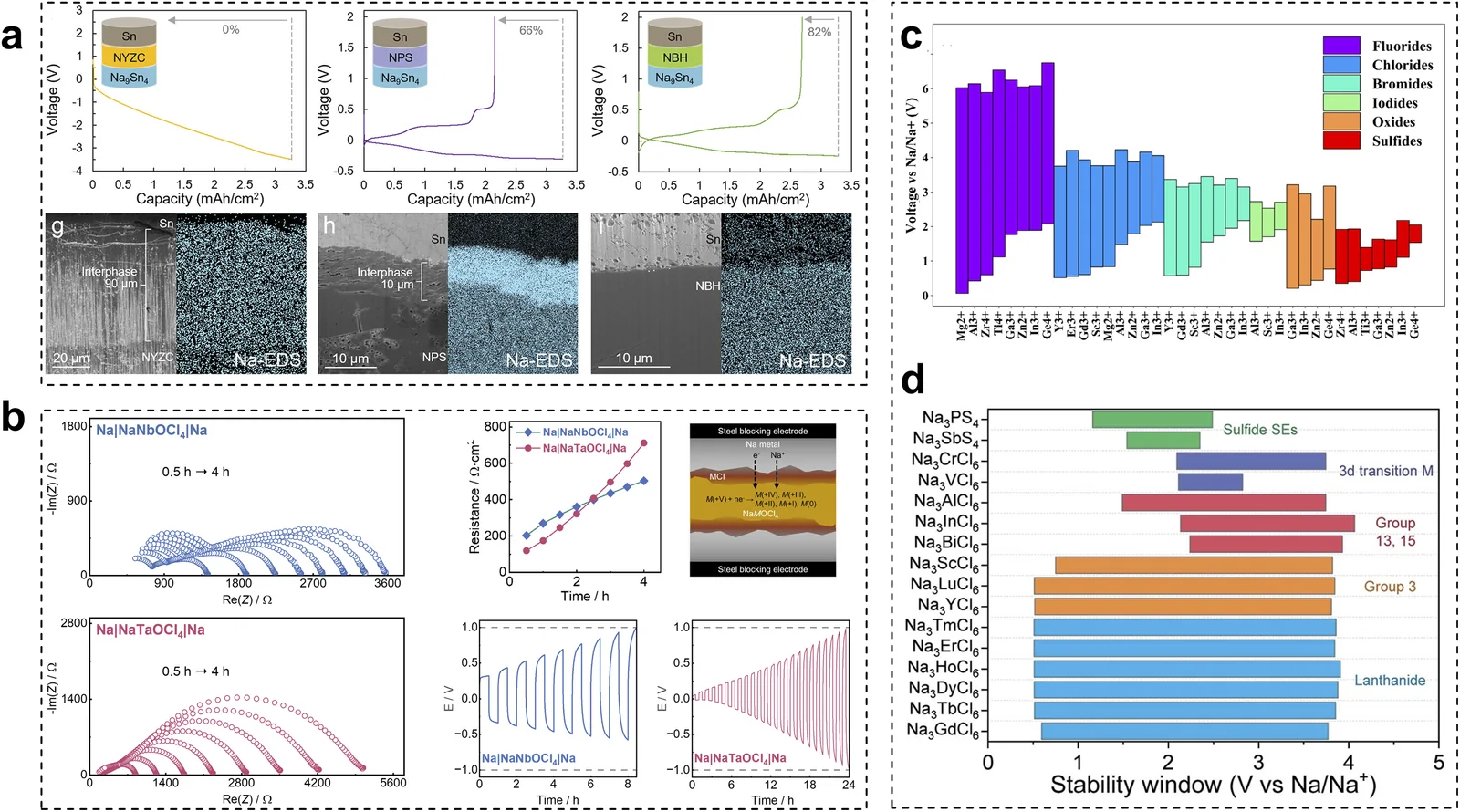
Reductive instability of sodium halide electrolytes at low-potential anodes. (a) Electrochemical and post-cycling interfacial evidence for SE decomposition against Na–Sn alloy anodes.^51^ Copyright 2022. *American Chemical Society*. (b) Impedance growth and mixed ionic and electronic conductor (MIEC) interphase formation at Na|NaMOCl_4_ interfaces.^52^ Copyright 2024. *American Chemical Society*. (c and d) Calculated electrochemical stability windows of representative sodium halides and sulfides.^94,116^ Copyright 2020. *American Chemical Society*. Copyright 2021. The Royal Society of Chemistry.

Spontaneous electron transfer from the anode to the electrolyte can be avoided only when the CBM or lowest unoccupied molecular orbital (LUMO) lies energetically above the Fermi level of the anode, corresponding to the Na/Na^+^ redox potential of approximately −2.71 V *vs.* SHE. In sodium halide electrolytes, the CBM or LUMO is mainly derived from the vacant d or s orbitals of high valent central metal cations, including the vacant 4d orbitals of Y^3+^, approximately −3.5 to −4.0 eV *vs.* SHE, the vacant 5 s orbitals of In^3+^, approximately −2.8 to −3.2 eV *vs.* SHE, and the vacant 4d orbitals of Zr^4+^, approximately −3.8 to −4.3 eV *vs.* SHE. Most of these energy levels are well below the Fermi level of metallic sodium, corresponding to an energetic offset of approximately 0.5 to 1.6 eV. This provides a clear thermodynamic driving force for electron injection from metallic Na into the halide lattice.^94^ DFT calculations further show that the lower limits of the electrochemical stability windows of Na_3_YCl_6_ and Na_3_YBr_6_ are 0.51 and 0.57 V *vs.* Na/Na^+^, respectively, both well above the 0 V redox potential of metallic sodium (Fig. 9c). These results confirm, from a first principles perspective, the intrinsic thermodynamic instability of sodium halides at the potential of metallic sodium.^94^ In addition, high throughput theoretical screening of 26 Na_3_MCl_6_ derivatives confirmed that the reduction limits of nearly all typical sodium ternary chlorides are higher than the Fermi level of metallic Na (Fig. 9d).^116^

In summary, anode side failure of halide SEs originates from their relatively low CBM or LUMO energy levels. This electronic structure enables electron injection from low-potential anodes, followed by the continuous evolution of mixed ionic and electronic conducting interfacial layers and, ultimately, dendrite penetration driven by coupled chemical and mechanical stresses. This cascading failure process constitutes one of the key intrinsic limitations of halide SEs in full cell engineering. It also defines a relatively clear application boundary: most sodium halide SEs are unsuitable for direct physical contact with metallic sodium or low potential hard carbon anodes. Therefore, resolving anode side reductive instability within a single halide SE framework remains fundamentally challenging. This limitation contrasts sharply with the high oxidative stability of halides at cathode interfaces. The same class of materials can exhibit a wide oxidative stability window approaching 4 V, yet undergo rapid interfacial destabilization below approximately 1.5 V on the reduction side. The underlying reason is that a single inorganic framework can hardly combine a deep VBM for oxidation resistance with a shallow CBM for reduction resistance. This electronic structure tradeoff does not mean that a universal sodium electrolyte is fundamentally impossible. In principle, a universal electrolyte would need to combine a deep VBM for oxidation resistance, a sufficiently high CBM for reduction resistance, fast Na^+^ transport, and adequate mechanical compliance over a broad Na chemical potential range. Achieving these coupled requirements within a single material remains a difficult materials discovery challenge, because lattice features that promote Na^+^ migration can also reduce the electronic stability margin or introduce reducible species. Accordingly, spatially decoupling oxidation resistance and reduction tolerance currently provides a practical design route. This principle forms the logical basis for sulfide and halide heterogeneous electrolyte architectures, which will be discussed systematically in the next section. Representative sodium halide and mixed anion electrolytes are compared in Table 2, including reported cathode side evidence, anode side evidence, and actual cell configurations. The comparison is intended to clarify application boundaries rather than to assign a universal stability ranking.

**Table 2:** Comparison of representative sodium halide and mixed anion solid electrolytes in terms of ionic conductivity, electrochemical behavior, electrode compatibility, and mechanical characteristics. Some parameters were not reported in the cited studies and are marked as not reported (NR)

Electrolyte	*σ* (mS cm^−1^)	Cathode	Cutoff voltage	Anode	Cycling conditions	Cycling performance	Fabrication pressure	Ref.
**Crystalline chlorides and bromides**								
Na_2_ZrCl_6_	0.018	NaCrO_2_	1.4–3.5 V	Na–Sn, Na_3_PS_4_ and borohydride layers	0.1C, 22nd	83.7%	370 MPa	121
Na_2.25_Y_0.25_Zr_0.75_Cl_6_	0.066	NaCrO_2_	1.7–3.4 V	Na–Sn, Na_3_PS_4_ layer	1C, 1000th	89.3%	370 MPa	17
Na_2.4_Er_0.4_Zr_0.6_Cl_6_	0.04	Na_0.66_Fe_0.4_Mn_0.5_Mg_0.1_O_2_	2.4–4.0 V	Na Sn, Na_3_SbS_4_ layer	0.01C, 10th	64%	380 MPa	53
NaTaCl_6_	3.3	NaCu_0.12_Ni_0.22_Fe_0.33_Mn_0.33_O_2_	2.3–3.8 V	Na_2_Sn, Na_2.9375_PS_3.9375_Cl_0.0625_ layer	70 mA g^−1^, 300th	90.0%	3 tons	122
NaAlCl_4_	0.0085	NaCrO_2_	2.0–3.5 V	Na_3_Sn, Na_3_PS_4_ layer	0.2C, 300th	82.0%	370 MPa	119
Na_1.625_Zn_0.625_Al_0.375_Cl_4_	0.015	NaCrO_2_	1.8–3.5 V	Na_3_Sn, Na_3_PS_4_ layer	0.1C, 4th	NR	250 MPa	98

**UCl3 type and multication halides**								
Na_0.7_La_0.7_Zr_0.3_Cl_4_	0.29	NaCrO_2_	2.0–3.4 V	Na_2_Sn, Na_3_PS_4_ layer	0.3C, 70th	88%	260 MPa	90
Na_0.6_Ta_0.2_La_0.8_Cl_3.7_F_0.3_	0.60	Na_0.8_Li_0.1_Ni_0.2_Mn_0.7_O_2_	2.6–4.3 V	Na_15_Sn_4_, Na_3_PS_4_ layer	0.1C, 100th	85.0%	450 MPa	123
NaLa_0.472_Ce_0.472_Ta_0.155_Nb_0.155_Zr_0.155_Cl_6_	>1	Na_3_(VOPO_4_)_2_F	2.4 to 4.3 V	Na_15_Sn_4_, Na_3_PS_4_ layer	0.3C, 600th	88.3%	370 MPa	124
Na_0.4_Ta_0.236_La_0.472_Cl_3_	1.38	NaCrO_2_	1.9 to 3.5 V	Na_15_Sn_4_, Na_3_PS_4_ layer	0.3C, 300th	89.7%	300 MPa	118
Na_1.4_Zr_0.4_Ta_0.6_Cl_6_	1.95	Na_3_V_2_(PO_4_)_3_	2.3–3.7 V	Na_15_Sn_4_, Na_3_PS_4_ layer	0.5C, 1000th	86.1%	370 MPa	117
Na_2.25_Yb_0.25_Zr_0.75_Cl_6_	0.029	NaCrO_2_	1.7 to 3.4 V	Na_2_Sn, Na_3_PS_4_ layer	0.1C, 260th	93.0%	400 MPa	82

**Oxychlorides and oxide derived electrolytes**								
NaNbOCl_4_	1.51	P2 Na_0.78_Ni_0.31_Mn_0.67_Nb_0.02_O_2_	2.0 to 4.0 V	Na_15_Sn_4_, Na_3_PS_4_ layer	0.5C, 250th	82.61%	300 MPa	42
NaTaOCl_4_	1.5	Na_0.67_Mg_0.5_Fe_0.4_Mg_0.1_O_2_	1.5–4.2 V	Na_15_Sn_4_, Na_3_SbS_4_ layer	C/30, 25th	Nearly 50%	375 MPa	52
Na_1.33_Ta(B_4_O_7_)_0.33_Cl_5.67_	3.1	NaCu_0.12_Ni_0.22_Fe_0.33_Mn_0.33_O_2_	2.3 to 3.8 V	Na_2_Sn, Na_2.9375_PS_3.9375_Cl_0.0625_ layer	0.5C, 800th	81.6%	300 MPa	103
TaCl_5_ plus 0.5Na_2_CO_3_ derived TC NCO	5.01	Na_0.67_Mn_0.4_Ni_0.3_Fe_0.15_Li_0.1_Ti_0.05_O_2_	2.5 to 4.0 V	Na_15_Sn_4_, Na_3_PS_4_ layer	0.5C, 300th	92%	300 MPa	43
0.5Na_2_O_2_ plus TaCl_5_ derived NTOC	4.62	Na_0.85_Mn_0.5_Ni_0.4_Fe_0.1_O_2_	2.5 to 3.8 V	Na_15_Sn_4_, Na_3_PS_4_ layer	0.1C, 500th	66%	∼300 MPa	41
NaAlCl_2.5_O_0.75_	1.33	Na_3_(VOPO_4_)_2_F	3.0–4.3 V	Na, NASICON layer	0.1C, 100th	93.1%	5 MPa	107
AOC-2NaCl	∼0.1 to 1	Na_0.83_[(Mn_0.75_Ni_0.25_)_0.9_Li_0.1_]O_2_	2.5–4.0 V	Na Sn, Na_2.9_PS_3.9_Cl_0.1_	0.1C, 60th	83.5%	300 MPa	108
NaAlCl_4_, Al_2_O_3_, and interfacial oxychloride composite, CSE0.75	0.13	Na_0.67_Ni_0.33_Mn_0.67_O_2_	1.9 to 3.9 V	Na_3_Sn, Na_3_PS_3.85_O_0.15_ layer	C/5, 150th	59%	370 MPa	93
0.6NaClO plus AlCl_3_ plus 0.175SeO_2_	2.03	NaNi_0.4_Fe_0.2_Mn_0.4_O_2_	2.4–4.15 V	Na_15_Sn_4_, Na_3_PS_4_ layer	0.3C, 480th	80%	∼400 MPa	110
NaCl, mesoporous α AlF_3_, and NaF heterostructure	0.0654	Na_3_V_2_(PO_4_)_3_	2.4–3.8 V	Na	0.65C, 300th	67.3%	280 MPa	111

**Mixed anion halides**								
0.6Na_2_S plus 1.4NaCl plus ZrCl_4_, NZCS0.6	0.753	NaCrO_2_	2.0–3.4 V	Na_2_Sn, Na_3_PS_4_ layer	0.3C, 1000th	83%	400 MPa	113
ZrO_2_ plus 2Na_2_ZrCl_5_F	0.021	Na_0.66_Ni_0.1_Co_0.1_Mn_0.8_O_2_	1.5–5.0 V	Na_3_Sn, Na_3_PS_4_ layer	0.1C, 100th	47.4%	370 MPa	125

**Heterogeneous and polymer composites**								
0.62[Na_0.75_Sm_1.75_Cl_6_] plus 0.38[NaTaCl_6_]	∼2.7	Na_0.85_Mn_0.5_Ni_0.4_Fe_0.1_O_2_	2.3–4.0 V	Na Sn, Na_3_PS_4_ layer	0.2C, 100th	91.0%	≈400 MPa	126
Na_2_ZrCl_6_ and PAN	0.121	Na_3_V_2_(PO_4_)_3_	2.5–4.0 V	Na	1C, 500th	85%	NR	127

## 4. Interface engineering strategies for all solid-state sodium batteries

The core conclusions of the preceding two sections reveal that neither sulfide nor halide electrolytes can be regarded as intrinsically thermodynamically stable against direct Na metal contact, although their decomposition pathways and practical stabilization routes differ. Hard carbon should be considered separately because its porous and electronically conductive structure creates distributed contact regions with the solid electrolyte. Its accessible surface area, defects, and surface functional groups influence electrolyte reduction, interphase formation, and irreversible sodium consumption. Hard carbon therefore requires a distributed interphase that blocks electron transport while maintaining Na^+^ conduction, rather than a protection strategy designed only for a planar metal interface.

Halides are favored in cathode composite layers because of their higher oxidation resistance. Some sulfide transport layers may be placed toward the anode only when electronically insulating decomposition products or artificial interlayers suppress continued reduction by blocking electron transport and maintaining mechanical integrity. Efficient Na^+^ transport across the sulfide halide interface requires coordinated chemical and mechanical regulation. Because currently available sulfide and halide electrolytes do not independently meet all requirements for practical full cells, the core engineering issue is not only how to optimize a single electrolyte, but also how to design heterointerfaces that enable complementary electrolyte components to work together spatially. Functionally partitioned architectures are not universally superior to a future universal electrolyte. Rather, they provide a practical current strategy for decoupling conflicting functions, provided that the heterointerface remains chemically stable and does not become rate limiting for Na^+^ transport. Guided by this framework, this chapter discusses cathode surface passivation and microstructural optimization, artificial interlayer design and chemical regulation at the anode interface, and the compatibility and transport mechanisms of sulfide–halide interfaces. The goal is to establish general engineering principles that link microscopic interfacial chemistry with macroscopic cell architecture.

### 4.1 Cathode interface engineering strategies

In ASSSBs, achieving chemical and mechanical compatibility between layered oxide cathodes (*e.g.*, NaCrO_2_, NaNi_1/3_Fe_1/3_Mn_1/3_O_2_, and P2 type Na_2/3_Ni_1/3_Mn_2/3_O_2_) and solid electrolytes is essential for realizing high energy density. Owing to the fundamentally different anion chemistries of SEs, sulfide systems are more susceptible to oxidative decomposition at the cathode interface because of the low oxidation potential of sulfide ions. In contrast, the high oxidation potential of chloride ions endows halide systems with intrinsic compatibility with cathode operating voltages. These two classes of SEs therefore face distinct interfacial challenges and require independent yet complementary strategies. For sulfide SEs, the central strategy is to suppress, or in some cases exploit, decomposition reactions through physical separation, compositional regulation, and interfacial design. For halide SEs, research efforts primarily focus on improving ionic conductivity and extending the oxidation stability window to higher potentials, while building on their inherent cathode compatibility. The following sections review recent strategic advances in these two electrolyte systems.

#### 4.1.1 Sulfide SEs and oxide cathode interfaces

Sulfide SEs, such as Na_3_PS_4_ and Na_3_SbS_4_, can achieve RT ionic conductivities of 10^−4^ to 10^−3^ S cm^−1^ and form extensive interfacial contact with cathode particles through cold pressing, which led to high expectations in early studies.^27,30,57,72^ However, the oxidative decomposition of the S^2−^ framework typically initiates below 2.5 V *vs.* Na^+^/Na, whereas layered oxide cathodes commonly operate above 3.0 V. Therefore, from a thermodynamic perspective, oxidative decomposition of sulfide electrolytes at the cathode interface is energetically favorable. This fundamental mismatch determines the overall direction of cathode interface strategies for sulfide systems. Because complete thermodynamic suppression of decomposition is difficult, such reactions must instead be kinetically restrained or, under specific conditions, redirected to form beneficial interfacial products. Following this logic, strategic development has evolved from improving interfacial wetting to regulating interfacial chemistry and, ultimately, redefining the functional role of decomposition products.

In sulfide composite cathodes, solution-processed electrolyte coating provides an effective route to optimizing solid state interfacial contact. By uniformly distributing the SE phase on the surface of cathode active material particles, as shown in Fig. 10a and b, the solid electrolyte/cathode active material (SE/CAM) interface can be reconstructed, thereby improving the continuity of Na^+^ transport pathways within the composite cathode. This concept is well matched with the solution processability of Na_3_SbS_4_. Owing to its solubility in methanol or water, Na_3_SbS_4_ can be uniformly deposited onto cathode particle surfaces through a solution process, converting the conventional point contact between powder particles into continuous surface contact. The improvement was substantial: interfacial impedance decreased from approximately 30 kΩ for the cold pressed powder mixing method to approximately 0.6 kΩ (Fig. 10c), while the full cell discharge capacity increased from approximately 3 mAh g^−1^ to 108 mAh g^−1^ (Fig. 10d), approaching the theoretical value obtained in liquid electrolytes.^47,128^ These results highlight an often neglected principle for sulfide systems: the quality of ionic contact between the electrolyte and the cathode, rather than the absolute electrolyte content or bulk ionic conductivity alone, often governs full cell performance. This strategy has also been surfaces. Structural and elemental mapping analyses showed extended to FeS_2_ cathodes. Kim *et al.* synthesized Na_3_SbS_4_ through a homogeneous aqueous reaction of Na_2_S, Sb_2_S_3_, and elemental sulfur, and subsequently used it to coat FeS_2_ particle that Na_3_SbS_4_ formed a continuous and uniformly distributed electrolyte layer on the FeS_2_ surface, with no significant side reactions occurring during coating. The FeS_2_ cell constructed with this coated cathode and a sodium tin alloy anode exhibited good reversible capacity and cycling stability.^87^ These findings further confirm that solution-processed coating can improve interfacial contact between sulfide SEs and cathode particles, providing an effective pathway for constructing continuous Na^+^ transport channels.

**Fig. 10:**
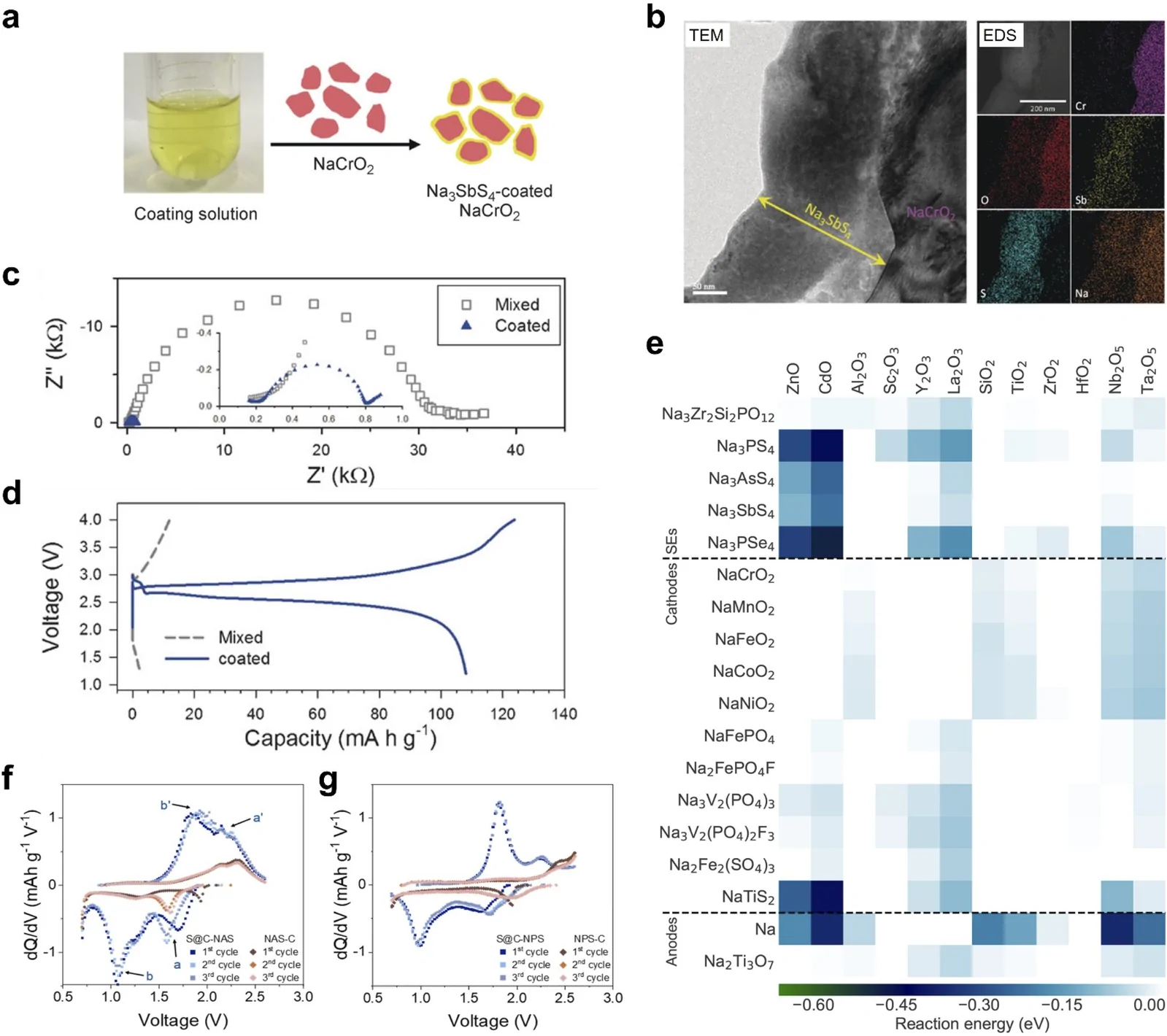
(a and b) Solution processed Na_3_SbS_4_ coating on NaCrO_2_ and corresponding microstructural characterization. (c and d) Interfacial impedance and initial voltage profiles of mixed and coated cathodes.^128^ Copyright 2016. Wiley VCH. (e) Computed reaction energies for oxide buffer layers with cathode active materials.^48^ Copyright 2017. *American Chemical Society*. (f and g) Redox pathway and differential capacity behavior of Na_3_SbS_4_/Na_3_PS_4_ sulfur cathodes.^50^ Copyright 2025. The Royal Society of Chemistry.

Similar physical isolation strategies have also motivated the search for inert buffer layer materials. First principles calculations, using chemical compatibility as a screening criterion, identified Sc_2_O_3_, SiO_2_, TiO_2_, ZrO_2_, and HfO_2_ as candidate coatings that are more compatible than conventional Al_2_O_3_, as illustrated in Fig. 10e.^48^ In another work, a solid gas reaction between cathode particles and gaseous P_2_O_5_ enabled the *in situ* formation of a NaPO_3_ nanolayer on the NaNi_1/3_Fe_1/3_Mn_1/3_O_2_ surface, demonstrating an active coating design concept. NaPO_3_ not only blocks direct electronic contact between sulfides and the cathode, but also promotes interfacial Na^+^ transport through the rotational motion of its polyanion groups, known as the paddlewheel mechanism. As a result, the capacity at 10C increased from 60 mAh g^−1^ for the unmodified material to 103 mAh g^−1^.^129^ This coating design, which integrates passivation and ion conduction, represents a clear advance beyond simple physical isolation.

A strategy that departs from conventional design frameworks redefines the cathode side function of sulfide SEs, shifting them from inert ion-conducting media to redox active components that can reversibly participate in cathode reactions. In a sodium sulfur ASSSBs based on Na_3_SbS_4_ (NSS), multi-modal spectroscopy, including *in situ* Raman spectroscopy, S K edge XANES, and Sb K edge EXAFS, confirmed the reversible self-redox mechanism of NSS. The SbS_4_^3−^ tetrahedral units remained structurally preserved during charge and discharge, allowing NSS to contribute simultaneously to ion conduction and capacity. The NSS sulfur cathode composite delivered a RT discharge capacity of 1976 mAh g^−1^, exceeding the theoretical capacity of elemental sulfur (Fig. 10f and g).^48,50^ The significance of this strategy extends beyond sodium sulfur batteries. It highlights a perspective that has long been underappreciated: redox activity of sulfides at the cathode is not necessarilydetrimental. Under appropriate structural constraints, deliberately designed and controllable redox reactions can become an integral part of interfacial stabilization.

Overall, cathode interface strategies for sulfide electrolyteshave evolved along a logical path from passive protection to active design. Physical isolation strategies, including inorganic coatings on cathode surfaces, oxide buffer layers, and organic polymer coatings, suppress oxidative decomposition by reducing direct contact between sulfides and high potential cathodes. Across multiple systems, these strategies have reduced interfacial impedance by several orders of magnitude and substantially improved cell capacity. Chemically regulated strategies, including entropy enhancement, cation and anion doping, and F^−^ assisted mechanical optimization, target the intrinsic composition of the electrolyte to improve air stability, processing tolerance, and ionic conductivity simultaneously. Building on these advances, the self-redox strategy for cathode side electrolytes further broadens the concept of interface stabilization. Controlled redox activity is not merely a process to be suppressed, but can also serve as an integral component of interface design. Future breakthroughs in sulfide-based cathode interface strategies may depend on integrating physical isolation and chemical regulation into a unified interfacial solution, as well as exploring additional routes to convert sulfide decomposition at the cathode side into functional interfaces.

#### 4.1.2 Halide SEs and oxide cathode interfaces

Recently, the transition from managing sulfide decomposition to exploiting halide stability changes the logic of cathode interface engineering. The strategy for cathode interface modification using halide SEs is rooted in a simple but crucial chemical fact: the oxidation potential of Cl^−^/Cl^0^ is significantly higher than that of S^2−^/S^0^, which generally gives halides a wider oxidative stability window. The oxidation limit of halides typically exceeds 3.8 V *vs.* Na^+^/Na, making them thermodynamically better matched with the operating voltage range of most layered oxide cathodes and substantially mitigating the oxidative decomposition problems encountered by sulfide SEs at high-voltage cathode interfaces.^37,78^ Goodwin *et al.* directly compared Na_3_SbS_4_ and Na_2.4_Er_0.4_Zr_0.6_Cl_6_ using Na_0.66_Fe_0.4_Mn_0.5_Mg_0.1_O_2_ as the cathode. The sulfide cathode composite showed a marked increase in interfacial impedance during charging and delivered a reversible capacity of only 7 to 15 mAh g^−1^, whereas the halide system achieved a reversible capacity close to the theoretical value and maintained more stable interfacial impedance.^53^ This comparison indicates that, for high-voltage cathode composites, interfacial chemical stability is often more critical than the pursuit of bulk ionic conductivity alone. Accordingly, stabilization strategies for halide cathode interfaces have mainly focused on cathode surface passivation, expansion of the oxidation stability window, and optimization of mechanical contact.

Although halide SEs generally exhibit a wide oxidation stability window, redox driven Cl^−^ and O^2−^ anion exchange may still occur between the halide SE and oxide cathode when the cathode potential exceeds 4 V (Fig. 11a). Such interfacial interdiffusion disrupts both the layered structure of the cathode surface and the Cl^−^ framework of the SE, leading to increased interfacial impedance.^130,131^ To address this issue, forming a thin NaF layer on the cathode active material surface has been demonstrated as an effective passivation strategy. XPS analysis indicates that the NaF coating suppresses Cl^−^ and O^2−^ exchange reactions.^131^ Moreover, the wide bandgap, electronic insulating character, and high chemical stability of NaF allow it to block electron transfer while facilitating Na^+^ transport across the interface under thin film conditions.^132^ Nevertheless, NaF should be viewed as a representative passivation layer rather than a universal solution. Inspired by high throughput computational screening strategies for cathode coating materials, similar thermodynamic compatibility criteria could be applied to sodium halide cathode interfaces to identify alternative passivation layers beyond NaF.

**Fig. 11:**
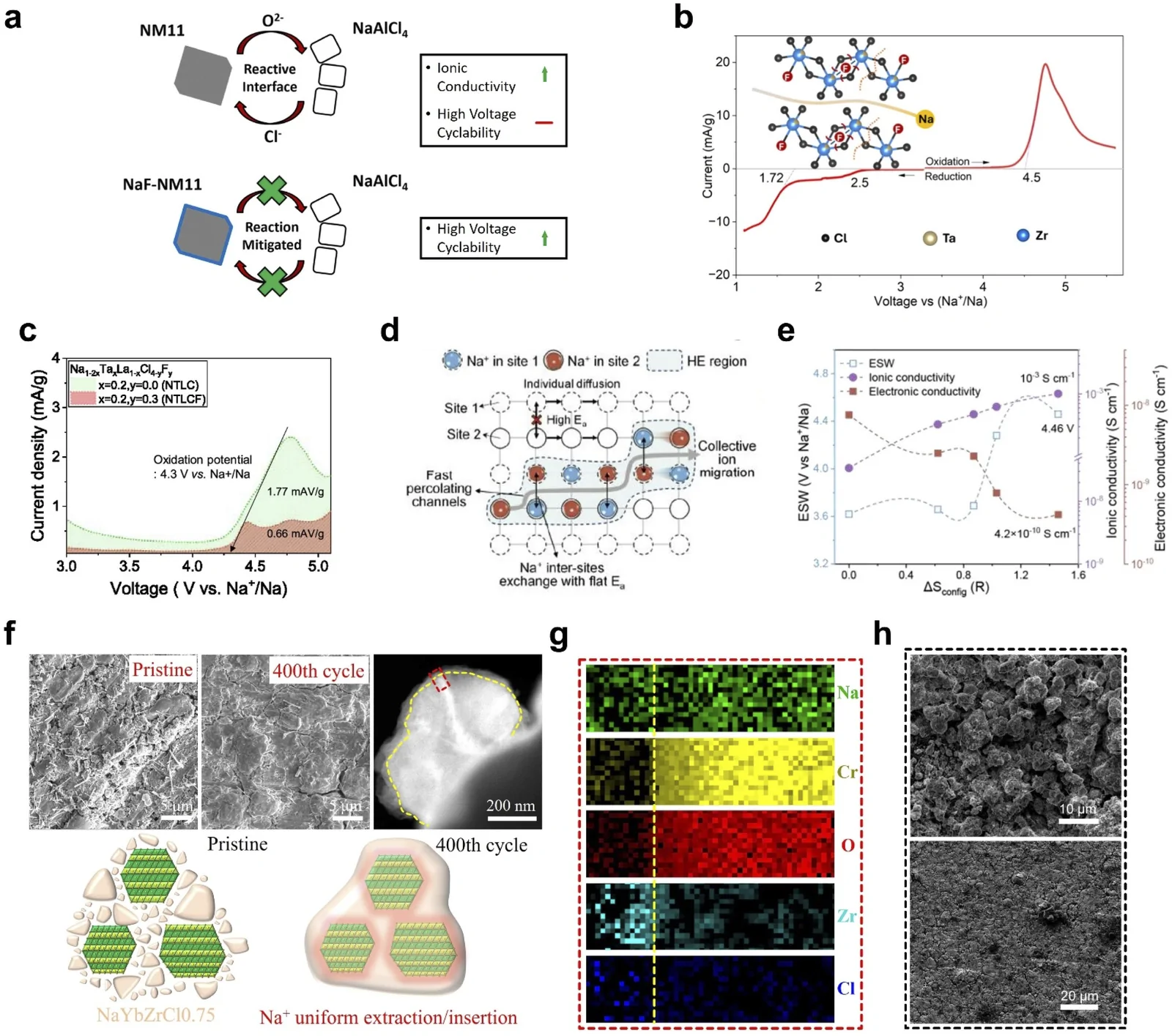
(a) Interfacial interactions in cathode composites.^131^ Copyright 2025. The Royal Society of Chemistry. (b and c) Oxidative stability of Na_1.4_Zr_0.4_Ta_0.6_Cl_6_ (NZTC).^133^ Copyright 2026. *American Chemical Society*. Oxidative stability of Na_0.6_Ta_0.2_La_0.8_Cl_4_ (NTLC), and Na_0.6_Ta_0.2_La_0.8_Cl_3.7_F_0.3_ (NTLCF) electrolytes from linear sweep voltammetry (LSV) tests.^123^ Copyright 2026. *American Chemical Society*. (d and e) Na^+^ percolation network and the relationship between configurational entropy, transport, and stability.^124^ Copyright 2026. Wiley VCH. (f and g) Microstructural and spectroscopic analysis of cycled Na_2.25_Yb_0.25_Zr_0.75_Cl_6_ (NaYbZrCl_0.75_) composite cathodes.^82^ Copyright 2023. Elsevier. (h) Morphology evolution of Na_1.4_Zr_0.4_Ta_0.6_Cl_6_ (NZTC) composite cathodes before and after cold pressing.^117^ Copyright 2025. *American Chemical Society*.

With regard to high-voltage cathodes operating above 4 V, another key strategy is to shift the oxidation limit of halides from approximately 3.8 V to higher potentials. The Cl^−^ and F^−^ dual anion framework Na_0.8_Zr_0.8_Ta_0.2_Cl_4.2−*x*_F_0.8+*x*_ (NTZ) also extends the oxidation stability to 4.5 V and exhibits an ionic conductivity of approximately 1 mS cm^−1^ under low stack pressure (Fig. 11b). Notably, the conductivity of this system increases as the stack pressure decreases, reaching 1.02 mS cm^−1^ at 74.5 MPa but decreasing to 0.6 mS cm^−1^ at 375 MPa. This anomalous behavior is attributed to the redistribution of amorphous free volume under low pressure, which favors Na^+^ coupled transport. A Na_3_V_2_(PO_4_)_3_ cathode based on this electrolyte retained 90% of its capacity after 300 cycles, while a Na_3_V_2_(PO_4_)_2_O_2_F cathode could be cycled up to 4.4 V and operated stably under a low stack pressure of 10 MPa.^133^ Fluorination stabilizes the anion framework primarily through the higher electronegativity of F^−^ and shorter metal halogen bonds, thereby suppressing Cl^−^ oxidation. The fluorinated UCl_3_ type chloride Na_0.6_Ta_0.2_La_0.8_Cl_3.7_F_0.3_ (NTLCF), in which F^−^ partially replaces Cl^−^ within the high coordination La based framework, was paired with the P2 type Na_0.8_Li_0.1_Ni_0.2_Mn_0.7_O_2_ high voltage cathode, enabling reversible oxygen redox at 4.6 V in an ASSSB (Fig. 11c).^123^ In contrast, reversible oxygen redox in liquid electrolytes becomes difficult to sustain above 4.2 V because of solvent oxidation.^123^ This result demonstrates that solid state halide SEs are not merely safer alternatives to liquid electrolytes, but may also provide a high voltage interfacial stability window that is difficult to access in liquid systems.^134,135^

In addition to fluorination, entropy enhancement can also improve the high voltage suitability of UCl_3_ type halides from the perspective of oxidation kinetics. The five cation compound NaLa_0.472_Ce_0.472_Ta_0.155_Nb_0.155_Zr_0.155_Cl_6_ belongs to the high entropy La and Ce based UCl_3_ type chloride family. In this material, high valent cations increase the kinetic barrier for Cl^−^ oxidation, while local structural distortions facilitate Na^+^ exchange hopping and entropy enhanced stability improves solvent tolerance.^124^ Together, these effects shift the oxidation limit to 4.46 V and enable 88.3% capacity retention after 600 cycles (Fig. 11d and e).^124^ Furthermore, by combining fluorination with a nanocomposite strategy, ZrO_2_ nanocrystals can be introduced into an amorphous Na_2_ZrCl_5_F matrix to form an oxygen substituted interfacial conductive phase, thereby constructing a halide nanocomposite electrolyte compatible with 5 V operation.^125^ These results demonstrate that improving the high voltage stability of halides depends not only on anion chemical regulation, but can also be further amplified through amorphous structure design and interfacial microstructure engineering.

Another important feature of many sodium halide SEs is their relatively low Young's modulus and greater deformability. First principles calculations show that representative sodium chlorides have low Young's moduli, such as 15.31 GPa for NaTaCl_6_, 17.68 GPa for NaAlCl_4_, and 21.65 GPa for Na_2_ZrCl_6_. Oxygen containing Al-based glasses provide an additional form of mechanical compliance. NaAlCl_2.5_O_0.75_ exhibits polymer like viscoelastic behavior, a storage modulus of approximately 3.2 GPa at 30 °C, and the ability to undergo rolling and thermal infiltration.^107^ These properties allow the electrolyte to fill voids within cathode composites and maintain contact under a stack pressure below 0.1 MPa. Therefore, the viability of Al-based oxychlorides is related not only to their enhanced ionic conductivity but also to their ability to form continuous cathode electrolyte contact under mild processing conditions. These mechanical properties allow halide particles to deform more readily under stack pressure, improve solid–solid contact with cathode particles, and help accommodate cathode volume changes during charge and discharge, thereby mitigating cycling induced interfacial contact loss.^46,136^ In the Zr^4+^ doped Na_3_YbCl_6_ system, lattice expansion and contraction of the NaCrO_2_ cathode generate a self-pressurization effect during cycling. This effect drives the soft chloride electrolyte to redistribute around cathode particles and progressively conform to their surfaces, gradually forming a uniformly coated core shell structure. This strategy does not require an additional cathode coating process, and a simplified producing process shows great potential in reducing the manufacturing cost of solid-state batteries. Instead, stable interfacial contact is enabled by three coupled factors: (1) cathode volume variation provides the mechanical driving force, (2) the deformability of the halide electrolyte allows structural adaptation, and (3) the favorable chemical compatibility between the electrolyte and cathode helps preserve the interface (Fig. 11f and g). Distribution of relaxation times (DRT) analysis further shows that the four DRT peaks of the halide cathode composite layer remain highly stable during long-term cycling, whereas the high-frequency interfacial peaks of the sulfide system continuously shift toward higher impedance. The corresponding full cell retained 74.1% of its capacity after 1000 cycles at 0.2C under RT conditions and 83.0% after 1900 cycles at 60 °C under 1C. These results provide proof of concept for improving interfacial contact by harnessing intrinsic cathode volume changes.^82^ Furthermore, studies have shown that the amorphous Na_1.4_Zr_0.4_Ta_0.6_Cl_6_ SE exhibits excellent densification behavior during cold pressing, enabling the formation of void free and compact contact layers. The high-density amorphous structure not only physically eliminates intergranular voids but also substantially reduces Na^+^ transport resistance by improving interfacial contact (Fig. 11h).^117^ Together with its intrinsic plastic deformability, NZTC can maintain continuous and stable contact with the cathode during cycling, thereby supporting long term electrochemical stability and interfacial integrity.

Overall, cathode interface strategies for halide systems follow a logical framework distinct from that of sulfide systems. Rather than primarily suppressing intrinsic decomposition, they focus on using high oxidative stability to enhance interfacial tolerance. Replacing sulfides with oxidation stable halides in the cathode composite layer is a fundamental interface stabilization strategy. Cathode surface passivation layers such as NaF can further suppress possible anion exchange and interfacial reactions under high voltage conditions. Fluorination, high-entropy modification, and nanocomposite strategies can extend the applicable voltage of halides beyond 4.5 V through anion bonding regulation, oxidation kinetic modulation, and microstructural engineering. In addition, the mechanical compliance of halides can improve solid–solid contact through a self-compression effect. Consequently, in practical battery architectures, a bilayer electrolyte design, using halides in the cathode composite layer and sulfides in the separator layer, represents a promising architectural approach for balancing cathode interface stability with overall ionic transport. Future development of halide cathode interface strategies will likely focus on low-cost material systems, improved anode side compatibility, and integrated electrode electrolyte architectures.

### 4.2 Interface design for sodium metal, alloy, and hard carbon anodes

#### 4.2.1 Sodium metal and alloy anodes

After the cathode side is addressed through electrolyte selection and interface regulation, the main compatibility challenge shifts to the sodium metal anode. Compared with cathode interfaces, where material-level compatibility can be improved by replacing sulfide electrolytes with halides of higher oxidative stability, anode interfaces present a more difficult challenge. Both sulfide and halide SEs are thermodynamically susceptible to reduction when directly exposed to metallic sodium. Therefore, constructing an interlayer between the sodium metal anode and the SE that combines high Na^+^ conductivity, low electronic conductivity, and chemical and mechanical robustness has become central to anode interface engineering.^51,137^ The concept of an artificial solid electrolyte interphase (SEI) originates from the spontaneous passivation of electrodes by electrolyte decomposition products in liquid systems. In ASSSBs, however, the lack of soluble precursors and dynamic repair pathways means that an effective SEI generally needs to be introduced through deliberate prefabrication.

From a design perspective, an ideal anode interlayer must satisfy several stringent and often competing requirements. It should provide sufficiently high Na^+^ conductivity (>10^−4^ S cm^−1^) to reduce charge transfer impedance, while maintaining extremely low electronic conductivity (<10^−8^ S cm^−1^) to suppress parasitic reactions. It must also remain thermodynamically stable against both metallic Na and the adjacent electrolyte, while being mechanically compliant enough to accommodate the repeated strain generated during Na deposition and stripping. In addition, its thickness should ideally be reduced to the submicron or even nanoscale range to minimize polarization losses.^51^ Three dimensional imaging based on *in situ* Raman spectroscopy and X-ray computed tomography further underscores the need for interface protection. Without a physical protective layer, reductive decomposition at sulfide interfaces, exemplified by the degradation of SbS_4_^3−^ polyhedral units, can propagate irreversibly into the bulk rather than remain self-limiting. The continuous growth of the reaction layer and the deterioration of electrode contact together indicate that a preformed artificial SEI is essential for stabilizing the anode interface.^15,49^ However, under these demanding conditions, a single phase material usually cannot fulfill all required functions. NaF offers excellent electronic insulation and chemical inertness, but its low intrinsic ionic conductivity can lead to substantial charge transfer polarization. By contrast, *in situ* alloy layers can provide fast ionic conduction pathways, but may undergo volume changes and mechanical degradation during repeated sodium insertion and extraction. These limitations of single component interlayers have driven the development of artificial SEI systems toward multiphase synergy and composite architectures.

Given these constraints, artificial SEI materials have evolved from single passivation phases into composite interfaces with complementary functions. Alloy interlayers can react *in situ* with metallic Na to form intermetallic compounds such as Na_15_Sn_4_ and Na_3_Sb, thereby creating a moderated Na chemical potential gradient at the interface and reducing the Na^+^ diffusion barrier. For example, amorphous passivation layers generated by the reductive decomposition of certain Na_3_SbS_4_-based compounds can provide partial interfacial self- protection. Nevertheless, alloy phases often experience large volume changes during cycling. Na_15_Sn_4_, for instance, can expand by approximately 420%, which may lead to particle pulverization and loss of mechanical contact. Therefore, alloy based interlayers generally require coupling with a mechanically robust second phase to maintain long term interfacial integrity.^138,139^

In contrast to alloy phases, which mainly facilitate rapid ion transport, NaF, NaCl, and closo type borohydrides primarily provide electron blocking and chemical passivation. NaF has a wide bandgap of approximately 11 eV and excellent chemical inertness, making it an effective component for suppressing interfacial electron leakage.^132,140^ However, because single phase NaF has limited Na^+^ conductivity and can induce charge transfer polarization, it is better suited as an electronically insulating scaffold in composite interlayers. A representative example is the spin coating of a THF solution of SbF_3_ onto a Na metal surface, which forms an approximately 10 µm thick bifunctional Na_3_Sb and NaF layer *in situ* (Fig. 12a–c). In this layer, NaF blocks electron transport, whereas Na_3_Sb provides fast Na^+^ transport pathways. Their microscopic functional complementarity enabled a Na_3_PS_4_ symmetric cell to cycle stably for more than 1000 h and allowed a full cell using a NaNi_1/3_Fe_1/3_Mn_1/3_O_2_ cathode to retain 88.3% of its capacity after 300 cycles at 0.2C.^49^ In addition, in a W and Cl co-doped sulfide network, an *in situ* precipitated NaCl rich insulating layer effectively blocks electron leakage (Fig. 12d–f).^58^ The closo type borohydride Na_4_B_10_H_10_B_12_H_12_ also shows good chemical compatibility on the anode side.^141^

**Fig. 12:**
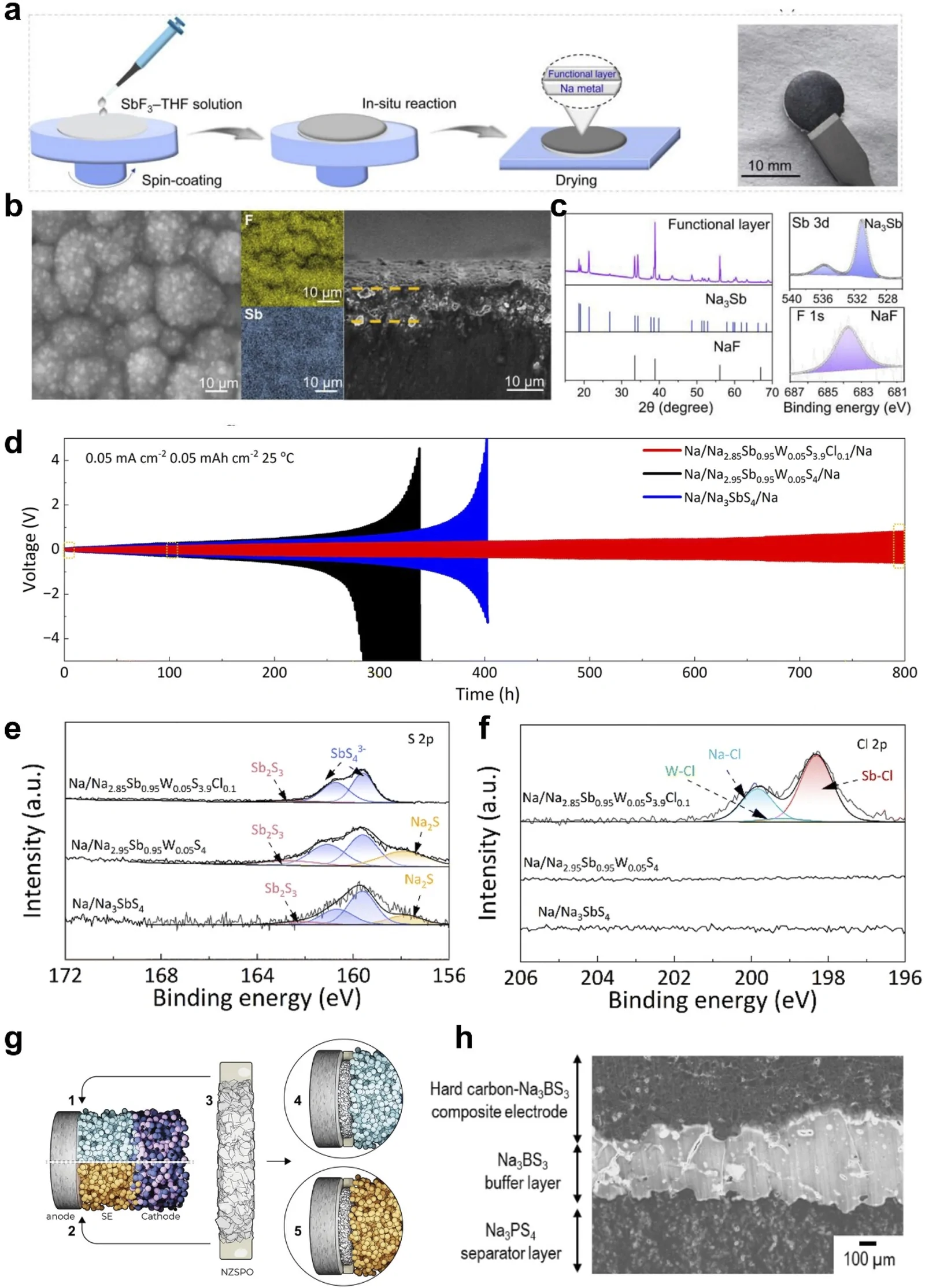
(a–c) XCT analysis of the Na_3_PS_4_|Na_3_Sb interface before and after Na plating or stripping, and SbF_3_-THF modified Na for stabilizing the Na_3_PS_4_|Na interface.^49^ Copyright 2026. Wiley VCH. (d–f) Cycling stability of sulfide electrolytes in Na symmetric cells and post cycling S 2p or Cl 2p XPS analysis.^58^ Copyright 2026. The Royal Society of Chemistry. (g) Na Sn anode and sulfide electrolyte interfaces with or without an Na_3.4_Zr_2_Si_2.4_P_0.6_O_12_ (NZSPO) interlayer.^142^ Copyright 2023. *American Chemical Society*. (h) Cross sectional SEM images of the cycled hard carbon/Na3BS3 composite anode with Na3BS3 buffer layers.^88^ Copyright 2023. The Author(s). Published by The Electrochemical Society of Japan.

In addition to *in situ* formed passivation phases, the pre-introduction of Na^+^ fast ion conductors as intermediate layers provides another effective route for anode protection. NASICON type ceramics, such as Na_3.4_Zr_2_Si_2.4_P_0.6_O_12_ (NZSPO), exhibit high electrochemical stability between Na and Sn alloy anodes and sulfide electrolytes (Fig. 12g).^142^ Na-β″-Al_2_O_3_ and NASICON type oxide ceramics generally possess a Na^+^ transference number close to 1.0, together with excellent electronic insulation, which helps suppress leakage currents associated with mixed ionic and electronic conducting networks. However, these ceramic interlayers are intrinsically brittle and have high Young's moduli, making them vulnerable to cracking under external pressure or electrode volume changes. To mitigate this mechanical limitation, polymer elastomers are often introduced as buffer phases, or high temperature cosintering is used to improve physical contact at the interface.^143,144^

Taken together, these developments suggest that multilayer gradient interfaces and organic inorganic composite interfaces have become important strategies for overcoming the functional limitations of single component interlayers on the anode side. Nevertheless, most current discussions and successful demonstrations of anode interlayer design remain focused on sulfide systems. For rapidly developing sodium-based halide SEs, reduction instability at low potentials has been recognized, but systematic experimental studies on artificial protective layers at the metallic Na interface remain scarce, largely because this material family is still emerging. By contrast, in lithium all-solid-state batteries, the compatibility between halide electrolytes, such as Li_3_YCl_6_ and Li_3_InCl_6_, and metallic Li has been more extensively investigated. These studies have provided useful design experience, including Li affine alloy primer layers, LiF and LiCl rich passivation interfaces, La based chloride SEs, and polymer barriers. The similar bandgap mismatch and microscopic electronic structure issues shared by sodium and lithium halide systems suggest that interface restructuring strategies developed for lithium halides may provide useful guidance for sodium halides. In particular, multifunctional intermediate layers should be designed to combine reduction tolerance, electron blocking capability, and stress accommodation, thereby broadening the practical operating window of halide solid state batteries. Although the lithium halide interface strategy provides a conceptual framework, the larger ionic radius of Na^+^ and the different coordination preferences mean that the structure and transport mechanism of the interface phase cannot be directly extrapolated, which means specific studies on sodium systems are required.

#### 4.2.2 Hard carbon anodes require distributed interphase design

Hard carbon is widely regarded as a practical negative electrode for sodium ion batteries because of its relatively low cost, practical reversible capacity, and limited volume variation. However, its interface in an all solid state battery differs from that of sodium metal. A sodium metal interface is approximately planar and is strongly affected by electrolyte reduction, sodium plating and stripping, contact loss, and dendrite penetration. Hard carbon instead provides a porous and electronically conductive network in which solid electrolyte particles contact numerous accessible carbon surfaces. Defects, edge sites, oxygen containing groups, and low potential sodium storage sites can contribute to electrolyte reduction and irreversible sodium consumption at different locations within the composite anode.

Direct evidence has been obtained for hard carbon and sulfide electrolytes. A hard carbon/Na_3_PS_4_ composite cell exhibited an initial irreversible capacity of 561 mAh g^−1^, with additional reaction features between 0.05 and 0.20 V attributed to the reductive decomposition of Na_3_PS_4_.^88^ Cross sectional analysis further revealed a Na rich reaction region at the boundary between the hard carbon electrode and the Na_3_PS_4_ separator. This observation is consistent with the formation of reduction products such as Na_2_S and electronically conducting Na_3_P. The latter can provide an electron transport pathway for continued electrolyte reduction. Replacing Na_3_PS_4_ with Na_3_BS_3_ only within the hard carbon composite reduced the initial irreversible capacity to 388 mAh g^−1^. When Na_3_BS_3_ was used both in the hard carbon composite and as buffer layers between the electrode and the Na_3_PS_4_ separator, the irreversible capacity decreased further to 122 mAh g^−1^. No obvious unfavorable reaction region was observed after cycling (Fig. 12h). These results show that both the electrolyte inside the composite anode and the separator boundary require protection. However, the reduced interphase growth should not be interpreted as complete thermodynamic stability.

The principal hard carbon interface objective is therefore to form a thin and electronically insulating interphase across the accessible carbon electrolyte contact area while maintaining Na^+^ transport. This approach should be combined with control of reactive surface groups and accessible porosity. Sodium inventory compensation may also be required when the initial irreversible capacity cannot be sufficiently reduced. NaBH_4_ based presodiation of hard carbon has increased the initial coulombic efficiency of an all-solid-state full cell to approximately 92%. In that cell, hard carbon contacted a closo borate electrolyte, whereas the halide electrolyte was used in the cathode composite. The result therefore supports presodiation as a sodium inventory strategy but does not establish direct hard carbon halide compatibility.

Ductile intermediate layers provide another route for improving physical contact. A comparison of Na(CB_9_H_10_)_0.7_(CB_11_H_12_)_0.3_ (NaCBH), NYZC, and a polymer electrolyte at the NASICON interface showed that NaCBH formed a lower resistance contact and enabled reversible operation of a hard carbon composite electrode. However, the hard carbon cell used NaCBH rather than NYZC, and the study did not demonstrate direct hard carbon/NYZC compatibility.^145^ A Na_2_ZrCl_6_/PAN composite membrane has also been used in a hard carbon full cell. Because the membrane contained an organic PAN matrix and the cell protocol included limited liquid electrolyte uptake, this result demonstrates the viability of an organic inorganic composite electrolyte rather than the intrinsic stability of dry Na_2_ZrCl_6_ against hard carbon.

Accordingly, an effective hard carbon interface should combine reduction tolerant electrolyte chemistry within the anode composite, an electronically insulating and Na^+^ conducting distributed interphase, controlled carbon surface chemistry and accessible porosity, and compensation for initial sodium loss where required.^127^ These requirements differ from a sodium metal interface, which must additionally maintain contact during plating and stripping and suppress dendrite penetration. Direct studies of dry hard carbon interfaces with pure sodium halide electrolytes remain an important research gap.

### 4.3 Interfacial compatibility between sulfide and halide electrolytes

The preceding discussion of sulfide oxidation at high voltage and halide reduction at low potential highlights a central materials challenge in ASSSBs: a single homogeneous electrolyte is unlikely to simultaneously satisfy the stability requirements of both a metallic Na anode and a high-voltage cathode operating above 4.0 V. In response, bilayer SE architectures have been developed to overcome the limited stability window of individual electrolytes. In such architectures, sulfides, which are relatively more compatible with the low-potential side, are placed adjacent to the anode, whereas halides, with higher oxidative stability, are incorporated on the cathode side.^13,51,53^ Preliminary evaluations of sodium based full cells indicate that this spatially separated electrolyte design is feasible and that the halide cathode side offers better interfacial stability at low rates than all sulfide systems.^53^ Recent Al-based oxychloride cells provide additional evidence for this functionally layered architecture. The AOC/2NaCl catholyte was combined with a Na_2.9_PS_3.9_Cl_0.1_ separator and a Na_15_Sn_4_ anode.^107^ The CSE0.75 catholyte was combined with a Na_3_PS_3.85_O_0.15_ separator and a Na_3_Sn anode, while NACDO was coupled with a Na_3_PS_4_ interlayer and a Na_15_Sn_4_ anode.^93^ These configurations use the Al-based electrolyte on the high voltage cathode side and a more reduction resistant electrolyte toward the anode. Their successful operation supports the functional separation of cathode and anode stability requirements. However, these cell results do not by themselves establish long term chemical stability or unrestricted Na^+^ transport across the Al oxychloride and sulfide interface.

However, when sulfide and halide electrolytes are tightly packed within a bilayer structure, the heterogeneous interface between them becomes the key factor determining the overall performance of the bilayer electrolyte. Unlike cathode–electrolyte or anode–electrolyte interfaces, both sides of the sulfide–halide interface consist of ionic conductors. Therefore, the core scientific issues extend beyond electrochemical decomposition to include interfacial chemical compatibility, inter-elemental diffusion, and the continuity of Na^+^ transport across the interface.

Similar instability at the sulfide–halide interface has been directly reported in lithium-based solid-state batteries. Relevant studies indicate that contact between these two types of SEs may lead to interfacial composition evolution, the formation of reaction phases, and ion transport hindrance.^137,147^ In contrast, research on sodium sulfide halide interfaces remains limited. Apart from the proof of concept work on Na_3_SbS_4_ and Na_2.4_Er_0.4_Zr_0.6_Cl_6_ bilayer or partitioned electrolyte systems (Fig. 13a–d),^53^ systematic studies on interfacial reaction thermodynamics, interdiffusion kinetics, structural evolution in the transition zone, and the mechanism of Na^+^ transport across the interface are still lacking. Recent work has further shown that a UCl_3_ type high coordination Sm or La based framework can be combined with amorphous NaTaCl_6_ to form a halide heterogeneous electrolyte with enhanced Na^+^ transport and high voltage stability.^126^ This result indicates that UCl_3_ type halides may be relevant to functionally partitioned architectures through their integration with complementary halide phases. However, Na_3_PS_4_ was still placed between the heterogeneous electrolyte and the Na Sn anode in the reported cell configuration. Therefore, this study should not be interpreted as evidence that UCl_3_ type halides are directly stable against Na metal or suitable as an anode contacting electrolyte without further interfacial protection. Direct sodium evidence for sulfide and halide heterointerfaces remains limited to a small number of proof of concept studies. Therefore, the following discussion of lithium systems is included as mechanistic guidance and should not be interpreted as direct experimental validation for sodium systems.

**Fig. 13:**
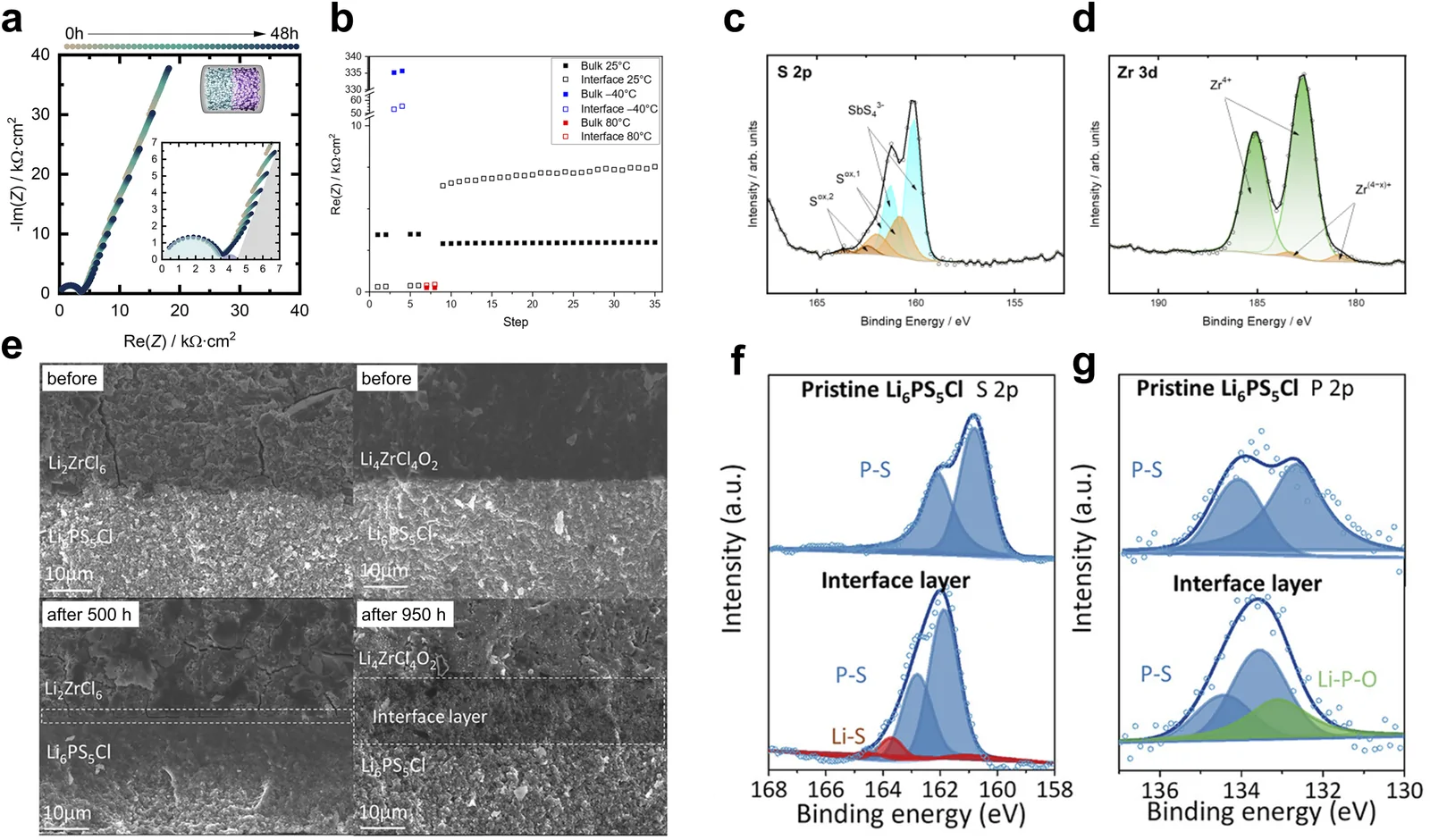
Interfacial compatibility between sulfide and halide SEs. Panels (a) to (d) present direct sodium evidence, including impedance evolution and chemical changes at the Na_3_SbS_4_|Na_2.4_Er_0.4_Zr_0.6_Cl_6_ interface.^53^ Copyright 2024. *American Chemical Society*. Panels (e) to (g) present lithium derived mechanistic insight into possible interfacial reaction pathways and passivation strategies. These lithium results are included as conceptual guidance and do not constitute direct experimental validation in sodium systems.^146^ Copyright 2025. Wiley VCH.

Studies on lithium systems have shown that the contact between sulfide and halide electrolytes is typically not fully thermodynamically stable. Due to differences in anion chemical potential, local coordination environments, and carrier concentrations on both sides, anions such as S^2−^ and Cl^−^ may undergo interfacial interdiffusion, thereby forming an interface transition zone with a gradual composition gradient.^137,147^ At the same time, differences in ionic chemical potential on either side may also induce the formation of a space charge layer, thereby altering the local ion concentration distribution and migration barriers.^81^ Whether the reaction products ultimately formed at the interface promote or hinder ion transport depends on their intrinsic ionic conductivity, electronic insulation, and spatial distribution at the interface. Therefore, heterogeneous interfaces often become the key rate-limiting step in bilayer electrolyte systems.

To address these issues, various interface stabilization strategies have been developed for lithium systems. An early approach involved introducing artificial buffer layers such as Li_3_PO_4_ and LiNbO_3_ to suppress interfacial side reactions and space-charge layer effects by creating an intermediate phase that is electrically insulating but ionically conductive.^21,139^ In recent years, interface engineering has evolved from the use of external buffer layers to a stage of adaptive interface stabilization. For example, in Zr-based lithium halides, high-concentration O^2−^ substitution to form oxygen-doped halides (such as Li_4_ZrCl_4_O_2_) not only enhances the bulk ionic conductivity but also enables a confined interfacial reaction upon contact with sulfides, leading to the *in situ* formation of an oxygen-rich passivation phase, Li_3_PO_4_, thereby mitigating continuous interfacial decomposition and impedance growth (Fig. 13e–g).^146^ This result demonstrates a lithium route for limiting interfacial decomposition through composition regulated passivation. For sodium systems, it provides a mechanistic hypothesis rather than an established design rule, and its applicability requires direct sodium specific verification. These strategies should be regarded as testable future hypotheses. For example, oxygen or sulfur incorporation into sodium halides may alter interfacial reaction pathways with Na_3_PS_4_ and may enable the formation of Na^+^ conducting transition layers. Whether such reaction products form, remain electronically insulating, and support continuous Na^+^ transport must be established by direct sodium experiments.

Specifically, three sodium specific factors should be considered: (1) the larger Na^+^ radius requires wider migration bottlenecks and greater local free volume; (2) mixed O^2−^, S^2−^, and Cl^−^ environments create more diverse Na^+^ coordination and site energy distributions; and (3) space charge behavior may differ because Na^+^ defect chemistry and interfacial chemical potential mismatch are not equivalent to those in lithium systems. Therefore, interface design principles established in lithium systems cannot be directly transferred to sodium systems.^20^ At present, the interdiffusion products formed between sodium sulfides, such as Na_3_SbS_4_ and Na_3_PS_4_, and sodium halides, such as Na_3_YCl_6_ and Na_2_ZrCl_6_, remain poorly understood. Their phase stability and Na^+^ transport mechanisms in mixed anion environments also require systematic investigation. These findings identify mixed sulfur chlorine anion systems as possible bridge phase candidates for sodium sulfide and halide interfaces. However, their phase stability, interfacial formation mechanism, and Na^+^ transport properties have not yet been systematically established. Incorporating S_2_^−^ into sodium halides and constructing compositionally graded sulfur chlorine interfaces should therefore be regarded as proposed future directions rather than experimentally validated sodium interface strategies.

In summary, bilayer SE architectures combining sulfides and halides provide a promising route for balancing high voltage stability with low potential compatibility in ASSSBs. However, their practical performance is ultimately governed by the chemical stability, Na^+^ transport continuity, and area specific resistance of the sulfide halide interface. At present, no sodium specific experimental or computational benchmark defines the maximum allowable area specific resistance of a sulfide and halide junction before it becomes rate limiting in a practical cell. Such a threshold cannot be universal because the allowable junction polarization depends on areal capacity, current density, temperature, stack pressure, total cell resistance, and the targeted voltage efficiency. Sodium systems remain at an early stage in this field, and systematic studies of interfacial reaction mechanisms, mutual diffusion behavior, resistance evolution, and the structure and transport properties of transition zones are still lacking. More importantly, the sulfide halide interface should not be viewed simply as a physical boundary between two materials. Instead, it should be regarded as an interfacial phase region with its own chemical composition, defect structure, and ion transport characteristics. Future studies should combine diffusion couple experiments, *in situ* characterization, first principles calculations, and cell level impedance analysis to establish phase diagrams for mutual diffusion between sodium sulfides and halides, theoretical models for interfacial evolution, and operating condition dependent resistance budgets for sulfide and halide junctions. Drawing on the progression of lithium systems from artificial buffer layers to adaptive interface engineering, future work should explore sulfur-doped halides, mixed-anion transition layers, and compositionally graded interfaces. As the understanding of interfacial chemistry and ion transport deepens, the development of all-solid-state sodium batteries is expected to shift from optimizing individual electrolyte properties toward architectural engineering centered on heterogeneous interface regulation.

## 5. Conclusions

In conclusion, for sulfide and halide SEs in ASSSBs, the electrolyte behavior is controlled by a common chemical logic: anion electronic structure governs band positions, band positions define oxidative and reductive stability limits, and the same anion framework also shapes Na^+^ migration through lattice softness, polarizability, and local coordination geometry. In sulfides, the large radius and high polarizability of S^2−^ produce soft lattices, wide Na^+^ migration channels, and low migration barriers, leading to high room temperature ionic conductivity. However, the high-lying S 3p-derived valence band also makes sulfides prone to oxidative decomposition at high-voltage cathodes. In halides, the deeper Cl-derived valence band provides stronger oxidation resistance and better cathode compatibility, but the low-lying conduction band associated with high-valent metal centers causes poor reduction stability near Na metal or other low potential anodes. Therefore, the key issue is not simply how to maximize bulk ionic conductivity or widen the electrochemical window, but how to balance ion transport, cathode oxidation stability, and anode reduction stability within a practical full cell. Al-based sodium halides represent a promising low cost branch of cathode side electrolytes because of the abundance of Al and the availability of NaCl and AlCl_3_ precursors. Their room temperature transport can be substantially improved through oxygen incorporation, amorphization, and heterointerface construction. Nevertheless, the high performance formulations may require additional oxygen precursors, mesoporous starting materials, or thermal treatment, while their reduction instability generally prevents direct use against Na metal. Within the present framework, Al-based halides are therefore most viable as catholytes, mechanically compliant cathode interface media, and components of layered electrolyte architectures rather than as universal stand alone electrolytes.

This understanding points to a necessary shift in research paradigm. Sulfides and halides should not be viewed as competing single electrolyte candidates. Instead, their complementary properties provide the chemical basis for functionally partitioned electrolyte architectures. In such architectures, halides are placed near the cathode to use their high oxidation resistance and cathode compatibility, while sulfides serve as fast Na^+^ transport layers or anode side components where the reduction interface is comparatively more manageable. This design is not a simple stacking of different materials. Rather, it spatially decouples bulk Na^+^ transport, cathode oxidation stability, anode reduction protection, and mechanical accommodation, assigning these functions to different but mutually compatible electrolyte components. The performance of this architecture will therefore depend not only on the properties of individual electrolytes, but also on the chemical, electrochemical, and mechanical stability of the interfaces between them.

Looking forward, four major directions should be focused to make progress in material design:

(1) Optimization of the preferred electrode interfaces of sulfides and halides. At the Na metal interface, sulfides are also thermodynamically prone to reduction. Their practical behavior depends on whether the reaction layer becomes electronically blocking and mechanically intact or evolves into an electronically conductive and mechanically damaged interphase. Although some sulfide interfaces may be more amenable to stabilization than many halide interfaces, direct contact between sulfides and Na metal can still induce thermodynamically favored reduction, electronically conductive products, and chemomechanical failure. Improving sulfide anode stability will require electron blocking interlayers, alloy buffer layers, artificial Na ion conducting passivation layers, and controlled formation of benign reduction products. Conversely, although halides show better cathode compatibility than sulfides, long term cycling can still involve anion exchange, transition metal migration, carbon related side reactions, and contact degradation. Further improvement of halide cathode stability should therefore focus on cathode surface coatings, fluorination, high-entropy regulation, low-cost chloride design, and mechanically compliant composite cathode architectures.

(2) Precise design of sulfide halide interphases. Sulfide halide heterointerfaces should be treated as active interfacial phase regions rather than passive physical boundaries. Mixed-anion transition layers, including S/Cl containing bridge phases, oxygen-modified halides, sulfur-doped chlorides, or graded sulfide–chloride interfaces, may reduce chemical potential mismatch, suppress unfavorable interdiffusion, and maintain continuous Na-ion transport across the interface.

(3) *In situ* and *operando* characterization of interfacial evolution. Solid electrolyte interface degradation often involves coupled redox reactions, elemental migration, space-charge redistribution, mechanical cracking, void formation, and dendrite penetration. Techniques such as *in situ* XPS, *operando* Raman spectroscopy, solid-state NMR, Time of Flight Secondary Ion Mass Spectrometry (ToF SIMS) depth profiling, cryogenic electron microscopy, and X-ray computed tomography will be important for tracking these processes under realistic electrochemical and mechanical conditions.

(4) Machine learning assisted interfacial phase diagram construction. Sodium sulfide halide interfaces involve complex mixed anion chemistry, defect distributions, and Na-ion migration pathways that are difficult to capture by empirical screening alone. Combining first principles calculations, high throughput thermodynamic screening, molecular dynamics, machine learning force fields, and experimental diffusion couple data could help predict stable interfacial phases, compatible mixed-anion compositions, and low-barrier Na-ion transport pathways.

Overall, the development of ASSSBs should move from optimizing isolated electrolyte materials toward designing integrated electrolyte architectures. A useful electrolyte does not need to perform every function alone, but must perform a specific function reliably within a spatially organized cell. By assigning fast-ion transport, cathode oxidation resistance, anode reduction protection, and mechanical buffering to different but compatible components, functionally partitioned sulfide–halide electrolyte architectures may provide a practical route toward high-energy-density, long-cycle-life, and safe ASSSBs.

## Author contributions

Lin Li: investigation, data curation and writing – original draft. Wenqian Tian, Miao Deng, Ziyu Lu: review and editing. Siwu Li, and Chuang Yu: conceptualization, supervision, funding acquisition, and review and editing.

## Conflicts of interest

There are no conflicts to declare.

## Data Availability

No new experimental data, computational results, or primary datasets were generated during the preparation of this manuscript.
